# Transcriptome-wide modulation of splicing by the exon junction complex

**DOI:** 10.1186/s13059-014-0551-7

**Published:** 2014-12-05

**Authors:** Zhen Wang, Valentine Murigneux, Hervé Le Hir

**Affiliations:** Institut de Biologie de l’ENS, (IBENS), 46 rue d’Ulm, Paris, F-75005 France; Inserm U1024, Paris, F-75005 France; CNRS, UMR 8197, Paris, F-75005 France; Current address: Departments of Immunology and Genomes & Genetics, Institut Pasteur, CNRS-URA 1961, Paris, France

## Abstract

**Background:**

The exon junction complex (EJC) is a dynamic multi-protein complex deposited onto nuclear spliced mRNAs upstream of exon-exon junctions. The four core proteins, eIF4A3, Magoh, Y14 and MLN51, are stably bound to mRNAs during their lifecycle, serving as a binding platform for other nuclear and cytoplasmic proteins. Recent evidence has shown that the EJC is involved in the splicing regulation of some specific events in both *Drosophila* and mammalian cells.

**Results:**

Here, we show that knockdown of EJC core proteins causes widespread alternative splicing changes in mammalian cells. These splicing changes are specific to EJC core proteins, as knockdown of eIF4A3, Y14 and MLN51 shows similar splicing changes, and are different from knockdown of other splicing factors. The splicing changes can be rescued by a siRNA-resistant form of eIF4A3, indicating an involvement of EJC core proteins in regulating alternative splicing. Finally, we find that the splicing changes are linked with RNA polymerase II elongation rates.

**Conclusion:**

Taken together, this study reveals that the coupling between EJC proteins and splicing is broader than previously suspected, and that a possible link exists between mRNP assembly and splice site recognition.

**Electronic supplementary material:**

The online version of this article (doi:10.1186/s13059-014-0551-7) contains supplementary material, which is available to authorized users.

## Background

Gene expression in eukaryotes is a highly regulated multi-step process. In the cell, nascent transcripts associate with various proteins to form ribonucleoprotein (RNP) particles. The composition of RNPs, specific of each transcript, changes during the successive stages of mRNA life cycle to orchestrate post-transcriptional regulation [1]. The exon junction complex (EJC) plays a central role in connecting different post-transcriptional events [2,3].

The EJC is a multi-protein complex which consists of four core proteins (eIF4A3, Magoh, Y14 and MLN51), serving as a binding platform for other nuclear and cytoplasmic proteins [4]. The EJC is deposited onto mRNAs during splicing 24 nucleotides (nt) upstream of exon-exon junction in a sequence-independent manner [5-8]. The assembly of EJC is tightly coupled to the splicing process [9,10]. The splicing factor CWC22 participates in the initial step of EJC assembly by recruiting eIF4A3 [11,12]. Pre-EJC core is assembled in active spliceosomes where eIF4A3 binds the Magoh/Y14 heterodimer, and the full EJC core is formed when MLN51 joins the complex at the late stage of splicing [10]. Studies of EJC binding sites in mammalian cells have revealed that EJC are differentially loaded onto different exon junctions of the same mRNA, both at its canonical binding sites (-24 nt position) as well as non-canonical sites [7,8,13]. Up to now, however, the mechanism that regulates differential deposition remains unknown.

Functionally, the EJC has been shown to play a role in several post-splicing events, including mRNA transport [14], translation [15-17] and surveillance by nonsense-mediated mRNA decay (NMD) [18,19]. There are several pathways that trigger NMD, and the best-described examples involve the EJC. Once deposited, the EJC recruits Upf3b in the nucleus and Upf2 in the cytoplasm. When a ribosome stalls at a premature termination codon (PTC) more than 50 nt upstream of an EJC, Upf1 joins the downstream EJC to form the surveillance complex, and triggers translation inhibition and mRNA degradation [18,19]. NMD not only functions to prevent the translation of aberrant mRNAs that arise from transcription and splicing errors, but some alternative splicing (AS) events can also exploit NMD to regulate their expression (AS-NMD) [20-22]. Around one-third of alternative spliced mRNAs has been predicted to contain PTCs in the open reading frame, and they are targets for NMD [23,24]. Notably, several splicing factors including SR and hnRNP proteins utilise AS-NMD to auto-regulate their own transcript level through a negative feedback loop [21,25,26].

In addition, the EJC has been shown to be required for the splicing of *mapk* pre-mRNA and other long intron-containing pre-mRNAs in *Drosophila* [27,28], and two recent studies revealed that EJC components contribute to the splicing of weak intron 4 of the *piwi* transcript in *Drosophila* [29,30]. In *Xenopus*, eIF4A3 is also required for accurate splicing of ryanodine receptor pre-mRNA [31]. In addition to constitutive splicing of introns, EJC components are involved in the regulation of apoptotic regulator Bcl-x alternative splicing in mammalian cells [32]. However, in this case, the binding of EJC components to the Bcl-x pre-mRNA is required but does not follow the conventional mode of EJC core binding to spliced mRNAs. To determine the extent by which EJC may influence splicing in human cells, we investigated the impact of the knockdown (KD) of EJC core components on endogenous alternative splicing patterns in a transcriptome-wide scale. Downregulation of EJC core components causes global changes in alternative splicing, as well as splicing of a subset of constitutive exons, indicating that they are involved in general splice site recognition. The splicing changes are specific to EJC core proteins and require EJC core integrity. The effect of EJC core components downregulation is compensated by different drugs affecting RNA polymerase elongation rate, strongly suggesting a link between mRNP assembly and transcription.

## Results

### Transcriptomic analysis in EJC knockdown HeLa cells

To evaluate the effect of EJC on mRNA processing on a global scale, we performed mRNA-seq experiments in HeLa cells in which the EJC core protein eIF4A3, Y14 and MLN51 were independently depleted with specific siRNA. In parallel, we performed mRNA-seq in HeLa cells treated with Upf1 siRNA, a key regulator for the NMD pathway. The knockdown (KD) efficiency for each protein was confirmed by western blot and qPCR analysis (Additional file 1A and B). We can achieve 40% to 60% depletion for eIF4A3 protein, whereas 70% to 80% of Y14, MLN and Upf1 proteins were depleted. Since Magoh and Y14 act as a dimer, depletion of Y14 also decreased Magoh protein level (Additional file 1A). Two biological replicate experiments were performed, and 86% of the reads can be mapped to human genome as unique reads (Additional files 1C and 2). In total, mRNAseq generated a combined 93.8, 64.2, 66.2, 73.1 and 47.7 million reads for KD of GFP, eIF4A3, Y14, MLN and Upf1, respectively (Additional file 2). The total numbers of reads per gene are highly correlated between the two replicate experiments (Additional file 1D).

We performed differential expression analysis using the DEseq package [33]. As expected, each KD sample showed a significant decrease in the expression of the corresponding gene (Additional file 3). We chose a read coverage of 10 (approximately RPKM = 30) as a cutoff to filter genes with low expression levels. Correlation analysis showed that the expression changes among different EJC component KDs are more correlated than that between eIF4A3 KD and Upf1 KD (Additional file 1E). DEseq identified 95 genes (1%) to be significantly changed in EJC KD samples compared with GFP control (adjusted *P* <0.05), with four genes consistently changed among all three EJC KD conditions (Additional files 3 and 4A). The small number of overlapping genes is most likely due to the incomplete KD, especially in the case of eIF4A3. In addition, the EJC core proteins could have functions outside EJC, and the number of genes that are expected to change in all three different protein depletion are low. Similarly, comparing genes that are significantly changed in any of the EJC KD (EJC union) with Upf1 KD showed five overlapping genes, all of which were significantly increased (Additional file 4B). The changes in expression in different KD conditions can be validated by qPCR (Additional file 4C). Taken together, significant changes in the expression of a small number of genes can be detected by EJC downregulation.

### Widespread splicing changes are identified in EJC knockdown cells

**Figure 1:**
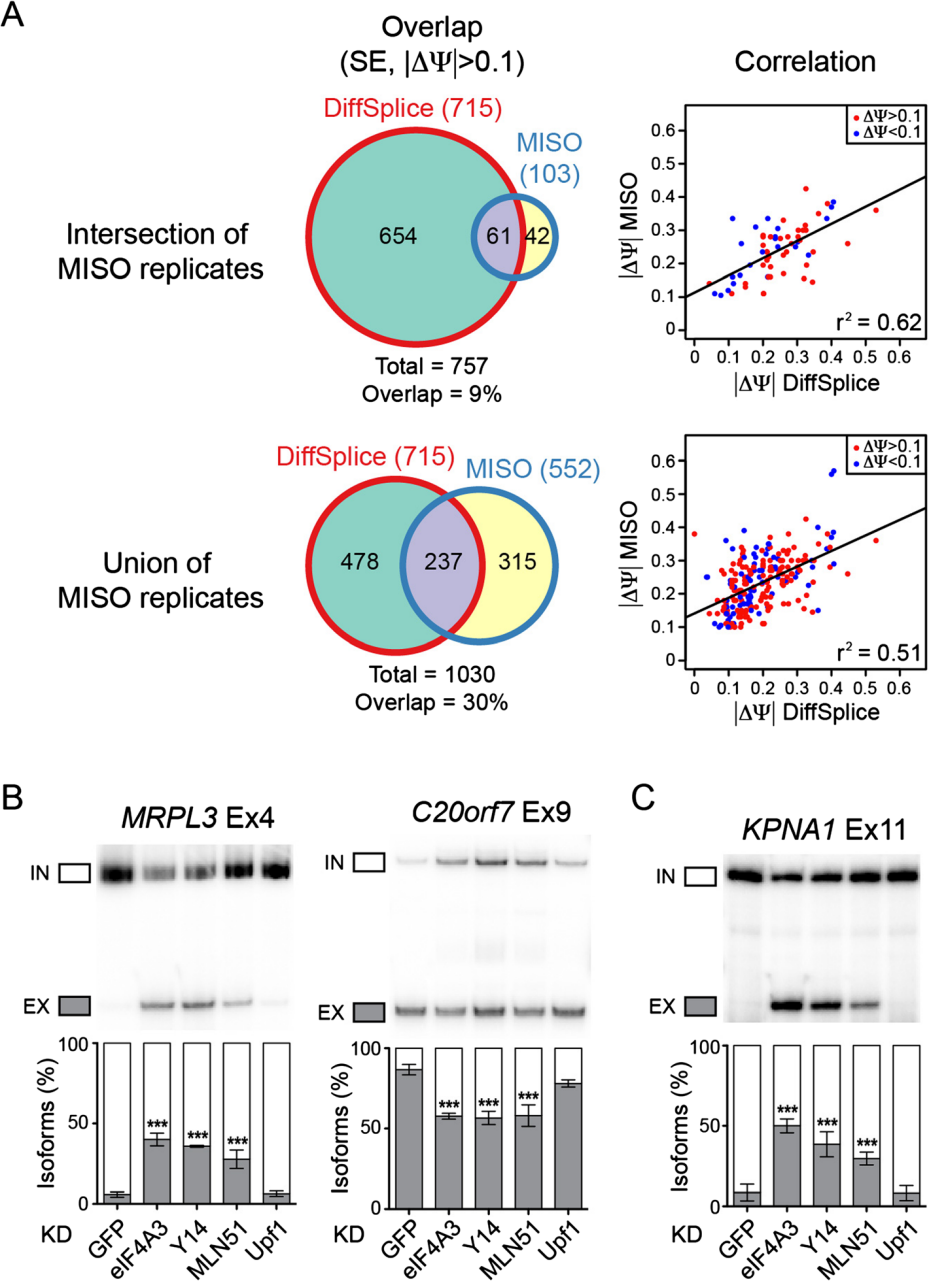
**Identification and validation of splicing changes in EJC KD cells. (A)** Venn diagrams showing the overlap between significant splicing events (cassette exons) identified with MISO and DiffSplice (left panels), using a threshold of 0.1 for ΔΨ. Correlation between the ΔΨ values predicted by MISO and DiffSplice are shown on the right, and Pearson’s correlation coefficient (r^2^) is indicated. **(B,C)** RT-PCR validation of two alternative exons (B) and one constitutive exon (C) in EJC and Upf1 KD cells. *MRPL3* is predicted to increase in exon skipping (ΔΨ >0.1) in EJC KD whereas *C20orf7* is predicted to increase in exon inclusion (ΔΨ <0.1) in EJC KD. *KPNA1* is predicted to increase in exon skipping (ΔΨ >0.1) in EJC KD. The quantification of triplicate experiments are shown below as mean ± SD. ***P <0.001, one-way ANOVA.

We analysed the differential exon usage in response to EJC proteins downregulation. To make a more comprehensive analysis, we used two different programs: MISO (Mixture-of-Isoform) [34] and DiffSplice [35]. MISO estimates differential isoform expression level from the known alternative splicing events. Since MISO does not handle replicates, each replicate experiment was analysed independently. DiffSplice, on the other hand, is an *ab initio* method that assembles the genome without known gene annotation and identifies alternative splicing modules where alternative transcripts diverge. This method allows the discovery of novel alternative splicing events as well as previously known alternative splicing events, and handles replicate experiments for sample variations. When we considered cassette exons with exon inclusion level significantly changed more than 0.1 (ΔΨ >0.1, 1 being the total abundance of all isoforms), MISO identified 103 or 552 cassette exons in any of the EJC KDs in both replicates (intersection of MISO replicates) or any of the replicates (union of MISO replicates), respectively (Figure 1A, Additional files 5A,B and 6). DiffSplice identified 715 cassette exons that have exon inclusion level changed more than 0.1 (ΔΨ >0.1) in EJC KD (Additional files 5C and 7). Among those cassette exons, 61 (9%) or 237 (30%) overlap with the ones identified by MISO, if we select genes changed in both or either MISO replicates, respectively (Figure 1A). The discrepancy between the two methods is partially due to the ability of DiffSplice to identify novel splicing events that are not within the MISO database. However, since DiffSplice does not assemble the genome according to previously known junctions, it does not identify some of the alternative events present in MISO. When looking at the cassette exons identified by both programs, the predicted exon inclusion change between MISO and DiffSplice is well correlated (Figure 1A). For validation, we chose the exons predicted by either MISO or DiffSplice or both programs, and selected a mixture of exons that are predicted to change in all EJC KD or in some of the EJC KD conditions. We validated 27 over 38 predicted cassette exon changes by RT-PCR (Figure 1B, 1C, Additional files 8 and 9). The validation rate for both programs are similar (77% for MISO and 80% for DiffSplice), and both programs may complement each other in identifying alternative splicing targets from mRNAseq data. Even though some of the alternative splicing changes were not predicted in some of the KD conditions (such as *TPCN1*), RT-PCR confirmed a significant splicing change for all EJC protein KDs, whereas UPF1 KD did not show the same splicing changes. Therefore, much more cassette exon events might be affected than those predicted by both programs.

Surprisingly, of the 715 cassette exon events identified by DiffSplice, 102 exons have no previous annotation in the genome as alternative exons. These could be novel, un-annotated alternative exons. Given that 91 (89%) showed an increase in exon skipping in EJC KD, most of these exons are normally constitutive exons in HeLa cells that are defectively spliced when EJC level was downregulated, demonstrating an important splicing activation of EJC on some splicing events (Figure 1C). Several of these differential splicing events, such as the *KPNA1* gene, can be validated by RT-PCR (Figure 1C, Additional file 9B). We decided to use the union of DiffSplice and the intersection of MISO replicates for further analysis (765 events, Additional file 10), which consist of both constitutive and alternative splicing events. Remarkably, a majority of cassette exons (487/765, 64%) showed an increase in exon skipping in EJC KD conditions, indicating that the EJC often facilitate exon recognition under normal conditions.

In addition, MISO and DiffSplice identified changes in alternative 5′ splice site (A5SS), alternative 3′ splice site (A3SS) and intron retention (IR) (Additional files 6 and 7). These events can also be validated by RT-PCR (9/10 were validated) (Additional files 8 and 11). Interestingly, MISO did not identify tandem alternative polyadenylation isoforms in any of the condition. Other splicing regulators have been shown to regulate tandem 3′UTR cleavage and processing by binding at the 3′UTR and blocking the access of the processing machinery [36]. Since EJC rarely binds 3′UTR, it seems that EJC does not influence alternative 3′ end processing and polyadenylation. Taken together, the mRNA-seq experiments detect splicing events reliably and accurately in response to EJC protein down-regulation, revealing the implication of EJC core proteins in the regulation of multiple splicing events.

### The splicing changes depend on EJC core integrity

**Figure 2:**
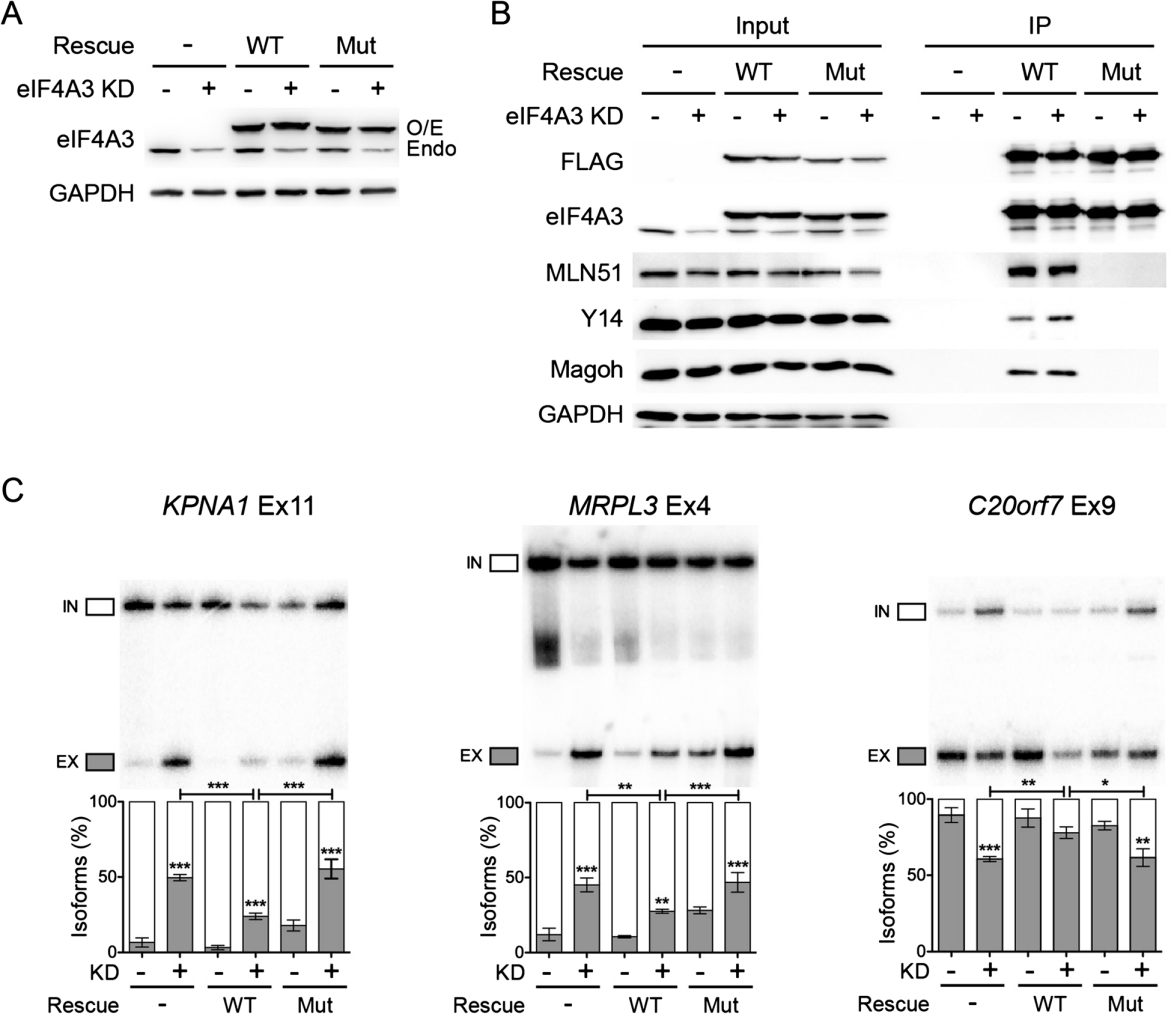
**The splicing changes in EJC KD can be rescued by overexpression of siRNA-resistant eIF4A3. (A)** Western blot showing the expression level of siRNA-resistant FLAG-eIF4A3 (WT) and a mutant that does not form EJC (D401KE402R, Mut) in the background of control and endogenous eIF4A3 KD condition. GAPDH is shown as a loading control. **(B)** Immunoprecipitation of FLAG-eIF4A3WT and FLAG-eIF4A3Mut in control and eIF4A3 KD cells. One/15th of input was loaded, and the immunoprecipitated samples were blotted for EJC components. GAPDH is used as a negative control. **(C)** RT-PCR of alternative splicing patterns in control and eIF4A3 KD cells with eIF4A3WT or Mut. Three representative genes were selected, with one constitutive exon (*KPNA1*) and two alternative exons (*MRPL3*, ΔΨ >0; *C20orf7*, ΔΨ <0). The quantification of triplicate experiments are shown below as mean ± SD. *P < 0.05; **P < 0.01; ***P < 0.001, one-way ANOVA.

A previous study showed that individual EJC components seem to preferentially associate with the Bcl-x pre-mRNA to regulate its alternative splicing, probably independently of EJC core integrity [32]. Therefore, we have examined whether the EJC-dependent splicing events identified in this study require EJC core. To this end, we performed rescue experiments with a siRNA-resistant form of FLAG-eIF4A3WT in the background of eIF4A3 KD cells. In parallel, we also transfected a mutant form of eIF4A3, FLAG-eIF4A3Mut that does not form EJC [10]. Comparing the effects of these two forms of eIF4A3 on EJC-dependent splicing is an important control to test EJC core integrity even if we cannot completely exclude that the mutant form of eIF4A3 can have some impact on alternative splicing independent of the EJC assembly. Both proteins can be overexpressed in HeLa cells at comparable levels whilst the endogenous eIF4A3 proteins were downregulated (Figure 2A). To control whether FLAG-eIF4A3WT can be incorporated into the EJC, we performed co-immunoprecipitation experiments with anti-FLAG tag. As expected, all three core proteins MLN51, Y14 and Magoh can be co-immunoprecipitated with the FLAG-eIF4A3WT but not with the mutant FLAG-eIF4A3Mut (Figure 2B).

Next, candidate splicing events were examined by RT-PCR under these conditions (Figure 2C, Additional file 12). For example, eIF4A3 KD caused an increase in exon skipping of the constitutive exon 11 of *KPNA1*. Overexpression of FLAG-eIF4A3WT significantly reduced the changes in exon skipping in eIF4A3 KD samples compared with control samples (Figure 2C). In contrast, overexpression of FLAG-eIF4A3Mut did not significantly reduce the changes in exon skipping in eIF4A3 KD samples (Figure 2C). Similar splicing patterns can be observed for the alternative exon 4 of *MRPL3* (Figure 2C). In the case of *C20orf7* exon 9, which increases in exon inclusion in eIF4A3 KD, the overexpression of FLAG-eIF4A3WT partially rescued the increased in exon inclusion in eIF4A3 KD cells whereas the mutant did not (Figure 2C). In addition, overexpression of the FLAG-eIF4A3Mut alone has a slight dominant-negative effect, even though it does not rescue the eIF4A3 KD cells (Figure 2C). This could be explained by FLAG-eIF4A3Mut competing with wild-type eIF4A3 and in turn causing less assembled EJC on mRNAs. Together, these results showed that EJC core integrity is important to regulate specific alternative splicing events.

### The splicing changes are not associated with differential decay rates

The splicing changes we observed could be due to differential decay rates of different isoforms. In mammals, around one-third of the alternative splicing are predicted to contain PTCs and may be subjected to AS-NMD [23,26]. Stop codons are considered as premature if they lie more than 50 nt upstream of an EJC. Decrease in EJC level could cause less efficient EJC-dependent NMD, and therefore indirectly increase the expression level of PTC-containing alternative spliced isoforms. In this case, the alternative splicing changes should also depend on Upf1 depletion. MISO and DiffSplice identified 58 (MISO, union of replicates) and 389 (DiffSplice) cassette exon changes in Upf1 KD, and only five (8.6%, MISO) and 158 (40.6%, DiffSplice) overlapped with EJC KD, indicating that an important proportion of the splicing changes identified with EJC KD are independent of NMD (Additional file 5B,C). Several splicing changes corresponding to Upf1 knockdown only, or both EJC and Upf1, were validated (Additional file 11D). We also searched for NMD features present in different isoforms for EJC-regulated genes. Among all the EJC-dependent splicing events, 155/451 (34.4%) was predicted to have a PTC in one of the isoforms. Out of the 155 exons, 23 (14.8%) showed an increase in total mRNA level (FC > 1.5) in EJC KDs, indicating that they are likely AS-NMD targets. Out of these, three are constitutive exons, and 10 are known to be NMD targets. These data showed that most of the splicing changes are not accompanied by changes in mRNA degradation, and that the EJC and Upf1 may affect alternative splicing profiles in different ways.

In the NMD surveillance complex, additional factors joins UPF proteins to function in NMD, including SMG1, SMG5, SMG6 and SMG7 [19]. Among these, SMG6 can interact with EJC [37] and is the catalytic endonuclease that cleaves the PTC-containing mRNAs [38,39]. If SMG6 is directly recruited by the EJC, some mRNA isoforms could eventually be degraded by SMG6 leading to a possible change in splicing patterns. To this end, we performed siRNA against SMG6, and checked alternative splicing pattern by RT-PCR (Additional file 13A). We selected both EJC-dependent alternative and constitutive exons, and KD of SMG6 or Upf1 showed no splicing changes (Additional file 13A). Therefore, the majority of alternative splicing changes we examined upon EJC KD are not linked to NMD.

The NMD pathway is not the only way to degrade RNA. The apparent alternative splicing patterns may be due to different stability of the isoforms. To test this possibility, we next performed a mRNA decay assay to measure mRNA levels when transcription was blocked by Actinomycin D. Cells were collected at different time points after adding the drugs, and the amount of alternative isoforms of several candidate genes were measured by RT-qPCR (Additional file 13B,C and 14). The *MYC* gene served as a positive control as it showed a rapid mRNA decay rate as previously described [40], whereas the negative control *DNM2* showed no decay after 24 h (Additional file 13B). We selected three candidate genes *MRPL3*, *HNRNPDL* and *PSMD2* to test our hypothesis. No significant changes in mRNA decay rate can be seen between control and eIF4A3 KD samples for the different isoforms (Additional file 13C). Therefore, the alternative splicing changes does not seem to be due to differential stability of the two isoforms.

### EJC core downregulation does not significantly affect splicing factors expression level

**Figure 3:**
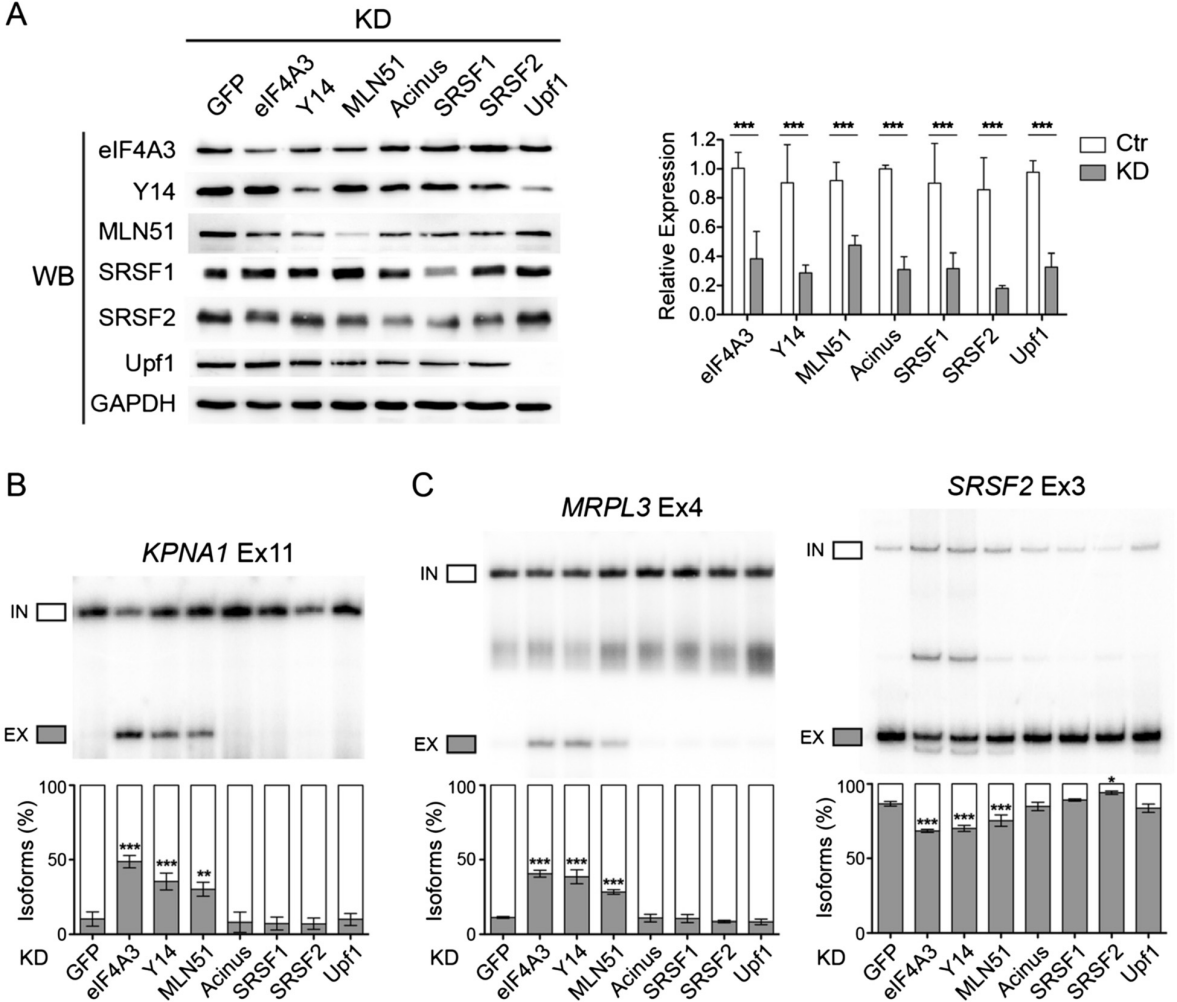
**EJC-dependent splicing changes are specific to EJC core protein. (A)** Western blot (left) and qPCR (right) showing the KD efficiency of EJC core proteins as well as EJC associated protein Acinus, and SR proteins SRSF1 and SRSF2. GAPDH is used as a control. **(B,C)** RT-PCR of splicing patterns of representative constitutive exons **(B)** and alternative exons **(C)** in different KD conditions. The quantification of triplicate experiments are shown below as mean ± SD. *P < 0.05; **P < 0.01; ***P < 0.001, one-way ANOVA.

Several EJC peripheral factors, including ASAP and PSAP complex components are *bona fide* splicing regulators [41,42]. In addition, EJCs associate with several SR proteins [7,8]. KD of EJC core components could prevent the association of these splicing factors to certain transcripts, causing changes in splice site choices. To test this, we downregulated Acinus, SRSF1 and SRSF2 with specific siRNAs in HeLa cells (Figure 3A). The EJC-dependent splicing events we tested were not affected by any of these splicing factors KD (Figure 3B and C, Additional files 9 and 11). This is true both for un-annotated cassette exons such as *KPNA1* and for alternative exons such as *MRPL3* and *C20orf7* when EJC KD promote exon skipping and inclusion, respectively (Figure 3B and C). An exception was the splicing changes in *SRSF2* exon 3, where SRSF2 downregulation caused the opposite effect of EJC KD (Additional file 9A). Another example is *BCAR1* exon 5 and 6, where KD of EJC caused a significant increase in exon skipping, whereas KD of Acinus and SRSF1 caused an opposite effect (Additional file 9B). Taken together, most of the splicing changes we examined are specific to the EJC core components, and are not caused by EJC-associated splicing factors.

The changes in alternative splicing could also be due to changes in expression of other general splicing regulators, and indirectly cause splicing changes. To this end, we examined the expression of several splicing regulators from the mRNA-seq data. We chose genes with the GO term mRNA processing and splicing regulation, and none of these genes were significantly changed upon EJC KD (Additional file 15). We also validated some splicing factors by qPCR based on their fold changes. Most of the genes were not significantly changed in all three EJC component KDs, indicating that the majority of the splicing changes we observed are most likely not caused by changes in splicing regulator expressions (Additional file 16). The exception is *DDX47,* which is significantly changed in all KD conditions (Additional file 16). DDX47 is a helicase involved in rRNA processing and its function in pre-mRNA splicing is currently unknown [43].

### EJC regulates cassette exons with longer flanking introns

We next analysed whether the EJC-dependent cassette exons have similar characteristics. We considered only cassette exons flanked by constitutive exons, and any exons with complex splicing patterns were excluded. Altogether, we defined 451 EJC-dependent cassette exons (293 exon ΔΨ >0, 65%; 158 exon ΔΨ <0, 35%). As control, we used 991 random alternative cassette exons without splicing changes in all EJC KD identified by MISO. In addition, we analysed the 73 EJC-dependent un-annotated cassette exons, and used 681 random constitutive exons without changes in EJC KD as a control.

**Figure 4:**
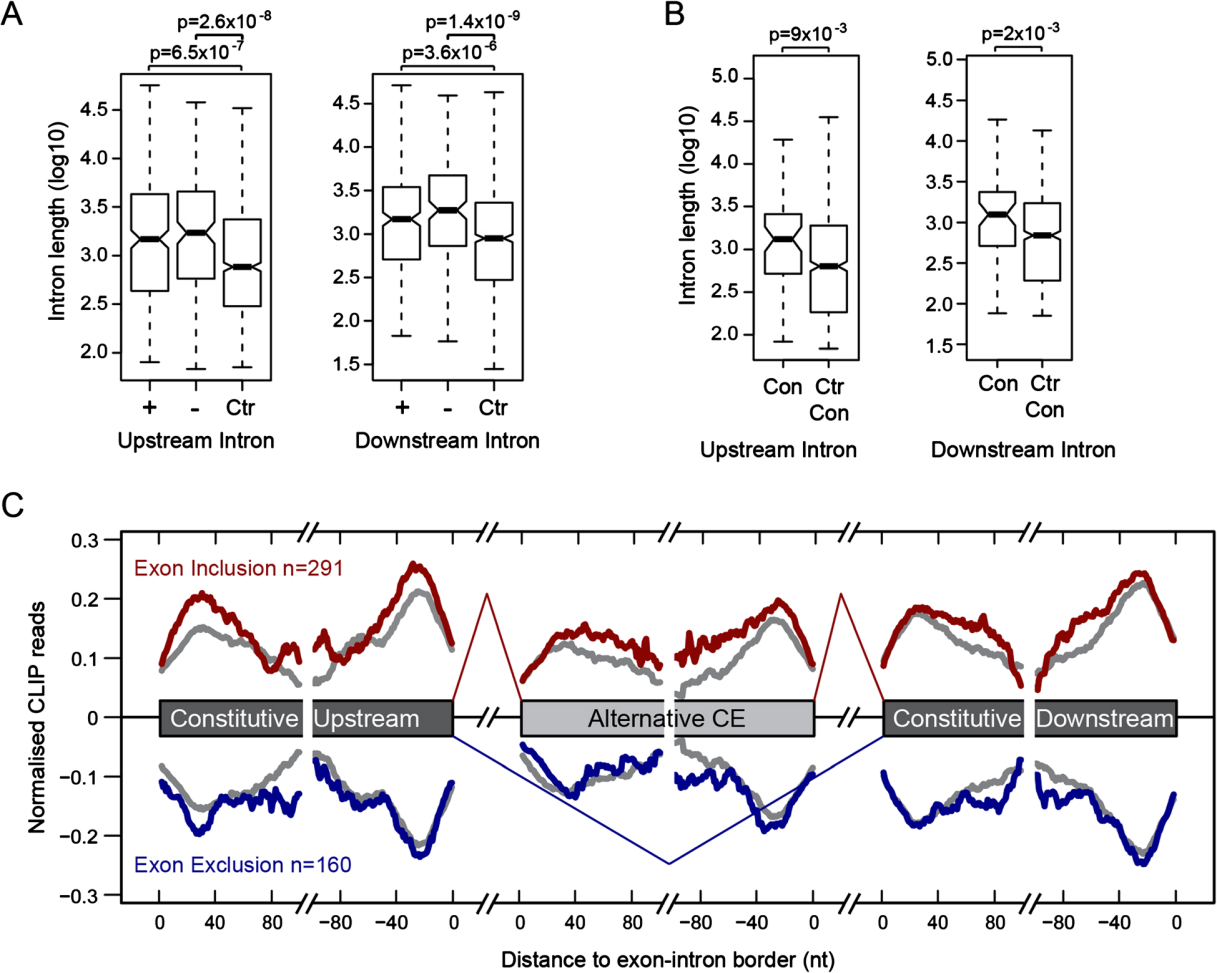
**Characteristics of EJC-dependent exons. (A)** Box plot of intron lengths for flanking introns, comparing EJC-dependent exon inclusion (+), exon skipping (−) and exons without splicing changes (Ctr). The *P* value is calculated with Mann–Whitney-Wilcoxon Test, and is indicated. **(B)** Box plot of intron lengths for flanking introns, comparing EJC-dependent constitutive exons (Con) and EJC-independent constitutive exons (Ctr Con). **(C)** RNA splicing map showing the normalised eIF4A3 CLIP reads in the upstream exon, cassette exon and downstream exon, 100 nt from the exon-intron border. EJC-dependent exon inclusion (+) are shown as red, exon skipping (−) are shown as blue, and control exons with has no splicing changes are shown as grey. The CLIP reads were normalised to mRNA expression level and exon length, and the height of CLIP reads at a position is the sum of all normalised CLIP reads at that position for each category of exons.

First, we examined the intron length. In *Drosophila*, it has been shown that the EJC components regulate the splicing of long-intron containing pre-mRNAs, and EJC KD reduced their expression more than short intron-containing pre-mRNAs [27]. We performed the same analysis with HeLa cell transcriptome data but found no correlation between intron length and differential expression level in any KD conditions in human cells (Additional file 17). We then analysed the introns flanking the alternative exons for EJC-dependent exon inclusion (ΔΨ >0.1, +), exon skipping (ΔΨ <0.1, −) and control exons (Figure 4A). The EJC-dependent cassette exons have significantly longer flanking introns on either side compared with the EJC-independent cassette exons, however the lengths are not different between exon inclusion and skipping events (Figure 4A). This is also true for the EJC-dependent constitutive exons, which have longer flanking introns compared with control (Figure 4B). Therefore, it seems that in human, the EJC also preferentially regulates alternative cassette exons that are flanked by longer introns.

We then analysed the exon length, splice site strength and the number of splicing enhancers and silencers (ESE, ESS, ISE, ISS) around the alternative and the flanking constitutive exons (Additional file 18). We observed more ESEs but less ISEs around the EJC-dependent alternative exons and flanking introns and exons, but there were no significant differences between EJC-dependent exon inclusion and exon skipping events (Additional file 18C and D). However, no specific ESEs were enriched (Additional file 19), indicating that ESE alone cannot predict whether an exon is EJC-dependent. When analysing the EJC-dependent constitutive exons, no significant changes can be seen in any of the features analysed (Additional file 17G-L). Therefore, the EJC-dependent cassette exons seem to be more heavily regulated through ESEs compared with the control exons, but no other specific feature can be correlated.

### EJC does not regulate splicing by binding at different positions of the exons

By integrating multiple independent genome-wide data sets, RNA splicing map has been used to show the positional regulatory effect of protein-RNA interactions [44]. For example, by combining Nova CLIP data and splice-junction microarray data, the RNA splicing map for Nova showed that it acts as a silencer when binding the upstream intron and within the exon, and acts as an enhancer when positioned at the downstream intron [44,45]. Here, we combined the CLIP-seq data of eIF4A3 [7] with the EJC-dependent splicing events to produce the RNA splicing map for EJC (Figure 4C). Since eIF4A3 binds mainly within the exon, we analysed the alternative exon as well as the flanking constitutive exon, 100 nt from either side of the exon/intron border. In the control exons (grey), eIF4A3 showed a characteristic enrichment of binding around −24 nt from the exon-intron border, and less well-defined enrichment in the 5′ half of the exon representing non-canonical peaks [7] (Figure 4C). Of note is that the CLIP signal is generally lower for alternative exons than for constitutive exons. For the EJC-dependent exon inclusion (red) and exon skipping (blue), the CLIP binding was similar to the control exons, with enrichment at both canonical and non-canonical positions (Figure 4C). We also generated RNA maps for EJC-dependent included constitutive (or un-annotated alternative) exons compared with control constitutive exons (Additional file 20A). The binding patterns were similar between control and EJC-dependent exons. It has been shown that EJC proteins bind the intron of *Bcl-x* to regulate its alternative splicing pattern. Therefore we also produced a RNA-map for introns flanking the cassette exon (Additional file 20B). Since eIF4A3 binds mainly in the exons, there is very little binding around the flanking introns, and there is no significant enrichment of binding around the EJC-dependent exons (Additional file 20B). Taken together, most likely the EJC does not act as other splicing regulators to regulate alternative splicing by binding at different positions.

### EJC regulates intron retention with shorter introns and weaker splice sites

In *Drosophila*, it has been recently shown that the core EJC components and the peripheral factor Acinus and RNPS1 can enhance the splicing of neighbouring introns [29,30]. If the EJC regulate splicing in a similar manner in mammalian cells, EJC downregulation would cause an increase in intron retention (IR) events. We therefore analysed the IR events predicted by MISO and DiffSplice. Overall, MISO and DiffSplice identified 134 events with increased intron retention upon EJC KD (ΔΨ <0.1, −), and 135 events with decreased intron level upon EJC KD (ΔΨ >0.1, +) (Additional file 21). We also identified 393 events in the MISO database that did not change in response to EJC KD. We performed the same analysis as for cassette exons. The EJC-dependent intron retention events have significantly shorter introns compared with control introns, but there is no significant difference between the EJC-dependent intron retention (+) and splicing (−) (Additional file 22A). We also found that the EJC-dependent intron retention and splicing events have significantly weaker splice sites, and the flanking exons have significantly less ESEs (Additional files 22B,C). None of the other features were significantly different from control intron retention events, indicating that the EJC-regulated intron retention and splicing events have generally weaker splicing potential, but whether they are spliced or retained seem to be a more complex decision.

### The splicing changes are linked with RNA polymerase II elongation

**Figure 5:**
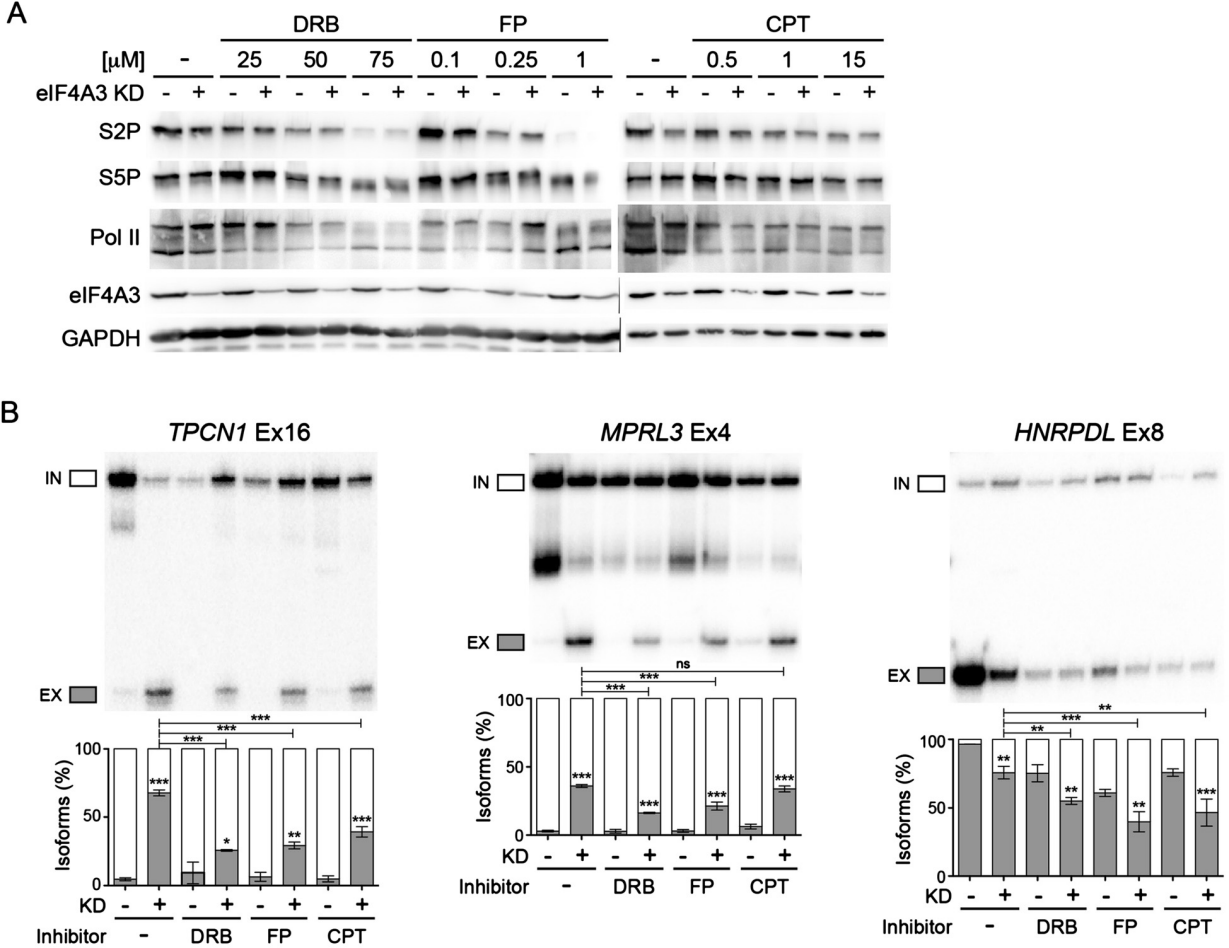
**EJC-dependent alternative splicing is linked with transcription. (A)** Western blot of control and eIF4A3 KD cells treated with different concentration of transcription elongation inhibitors (DRB, FP and CPT) for 16 h. GAPDH is used as a control. **(B)** RT-PCR of splicing patterns of representative exons in cells treated with different inhibitors. The quantification of triplicate experiments are shown below as mean ± SD. *P <0.05; **P <0.01; ***P <0.001, one-way ANOVA.

The different post-transcriptional steps are tightly coupled to transcription [46]. For example, changing Pol II elongation rate have an impact on global alternative splicing patterns [47]. Generally speaking, a slower Pol II elongation rate results in an increase in exon inclusion by extending the time window for splicing factors to recognise the weaker alternative splice sites [47,48]. We tested whether the EJC-dependent splicing events are linked with changes in transcription. To this end, we treated control and eIF4A3 KD cells with transcription elongation inhibitors: DRB (5,6-dichloro-1-β-D-ribofuranosyl-benz-imidazole) and Flavopiridol (FP). Both drugs inhibit the elongation factor P-TEFb [49,50], leading to decreased Ser2 phosphorylation of the Pol II CTD, and inhibition of transcription elongation [51,52]. Treatment with both drugs caused a decrease in Ser2 phosphorylation in control and knockdown cells, whereas Ser5 phosphorylation was only affected to a lesser extent (Figure 5A, Additional file 23A). Furthermore, the decrease in Ser2 phosphorylation was bigger with increasing concentration of drugs (Figure 5A). Western blot of Pol II confirmed a reduction in phosphorylated isoform (upper band) whereas the non-phosphorylated isoform (lower band) remains the same (Figure 5A). We also used camptothecin (CPT), which inhibits topoisomerase I and creates topoisomerase I-DNA adducts to block Pol II elongation [53,54]. Treatment with CPT did not change the phosphorylation level of Pol II CTD (Figure 5A). To evaluate if the drug treatment was efficient, we checked the alternative splicing of *CHD2* pre-mRNA, which increase exon skipping with CPT treatment [55] (Additional file 23C). All drug treatments caused an increase in exon skipping for CHD2, while eIF4A3 KD showed no effect (Additional file 23C).

We next monitored the effect of these drugs on several EJC-dependent splicing events by RT-PCR. For examples, the constitutive *TPCN1* exon 16 increased in exon skipping in eIF4A3 KD samples. Remarkably, the exon skipping level was reduced upon treatment with DRB, FP and CPT compared to control cells, while the drugs had no significant effect on the basal splicing pattern in control siRNA treated cells (Figure 5B). In this case, DRB had the biggest effect whereas CPT showed less decrease. For the alternative *MRPL3* exon 4, both DRB and FP reduced the exon skipping level caused by eIF4A3 KD whereas the effect of CPT is very weak (Figure 5B). In the case of *HNRPDL* gene, KD of eIF4A3 caused an increase in exon inclusion (Figure 5B). Addition of all inhibitors caused a further increase in exon inclusion not only in control samples, but also in eIF4A3 KD samples (Figure 5B). This increase in exon inclusion in control samples with elongation inhibitors has been shown before for *HNRPDL* [47], confirming that the splicing changes are due to a change in Pol II elongation. Therefore, several EJC-dependent splicing changes are linked with transcription elongation and Pol II phosphorylation.

To further confirm that the effect of EJC-dependent splicing changes are linked with transcription elongation rather than due to unspecific effects, we examined the *in situ* transcription rate of candidate genes in response to EJC KD using Padgett’s protocol [56]. In this procedure DRB is used to reversely block gene transcription *in vivo*, and after its removal, RT-qPCR was performed to assess the appearance of newly synthesised pre-mRNA. Two sets of primers corresponding to the beginning and the end of the gene were used, and the differences in time between the recoveries of expressions should indicate the relative speed of Pol II transcription elongation [56]. As expected, the positive control gene, *ITPR1*, showed an increase in pre-mRNA of exon 1 early after DRB removal (10 min), whereas exon 5, which is 133 Kb away, showed an increase much later (50 min) (Additional file 24A), in agreement with previous study [56]. In eIF4A3 and Y14 KD samples, the amount of pre-mRNAs of exon1 is higher after DRB removal, which could be due to an increase in Pol II speed or initiation. However, the similar amount of pre-mRNA accumulate at intron-exon5 argue for a change in transcription speed (Additional file 24). We also tested one of our candidate genes, *GLRX3* (Additional file 24). The exon 2 showed a recovery in 20 min in control cells, but only 10 min in eIF4A3 KD cells and even faster in Y14 KD cells, indicating an increase in transcription rate (Additional file 24). Furthermore, the recovery of exon 10 is around 30 min in control cells (10 min lag time), but faster (5 min lag time) in eIF4A3 KD cells (Additional file 24). Therefore, the reduced EJC level seems to increase Pol II transcription rate in some EJC-dependent genes, further demonstrating a link between some EJC-dependent events and Pol II transcription.

## Discussion and conclusions

Here, we present evidence that the EJC core proteins can affect a large set of alternative splicing changes in mammalian cells. Several studies before have indicated that the EJC protein can regulate splicing in different species [27-29,31,32]. However, these studies only focused on a specific set of candidate genes. To what extend the modulation of the expression of EJC components effect on alternative splicing holds true on a genomic scale, and whether the EJC is directly involved in regulating alternative splicing remains largely unknown. We analysed the transcriptome of human cells in which the EJC core proteins have been downregulated to study the alternative splicing changes related to EJC proteins. The reduction of EJC caused large numbers of alternative splicing changes. Different types of alternative splicing patterns were affected, with a majority of the cassette exons excluded in EJC KD. Interestingly, our study also reveals that the EJC KD affected the recognition of un-annotated cassette exons with 100% inclusion under normal growth conditions. Therefore, the EJC has a broad impact on splicing by contributing to the recognition of numerous exons under normal splicing conditions.

Using different computational tools combined with direct measurement of specific splicing isoforms, we discovered hundreds of splicing events that were similarly affected by the independent KD of three EJC core components. The EJC could differentially affect the stability of splicing isoforms via the coupling between alternative splicing and NMD (AS-NMD). However, this is not always the case because EJC KD did not influence the decay rate of the EJC-dependent spliced mRNAs that we have tested. We also investigated different ways in which EJC could regulate splicing more directly. An obvious explanation would be that these downregulations somehow affect the expression of one or several splicing factors, which in turn would affect a large number of splicing events. This cannot be completely excluded but our analysis of the expression of numerous splicing regulators did not detect candidates whose expression was affected by all three KDs independently. Another possibility would be that the EJC proteins bind specific pre-mRNAs and interfere with their splicing as previously observed for *Bcl-x* gene, which depends on eIF4A3 and Y14 as well as auxiliary splicing factors of Acinus, RNPS1 and SAP18 [32]. However, among all the splicing events affected by the individual EJC core components, a significant proportion depends on assembled EJC core. This is supported by the fact that overexpressing a mutant eIF4A3 that is unable to form the EJC core does not restore most of the EJC-dependent splicing events.

So far, pre-mRNA splicing is the only known process able to assemble the EJC core. Thus, how does a fully assembled EJC regulate alternative splicing, given that it is deposited and assembled after the splicing decision has been made [9,10,57]? The vast majority of mammalian pre-mRNAs contains multiple introns. Once assembled onto an exonic junction, the EJC and its associated factors could serve as a splicing regulator in the recognition of neighbouring splice sites, and in turn modulate the splicing pattern. In fact, recent studies in *Drosophila* have shown that the splicing of some specific weak introns is dependent on EJC components [29,30]. For example, splicing of intron 4 of the *piwi* transcript requires the flanking exon junctions and EJC core components as well as peripheral factors RNPS1 and Acinus [29,30]. Even if we cannot exclude the possibility that this attractive model is responsible for some particular splicing events in mammalian cells, several data argue against this as a general mechanism. First, we downregulated EJC associated splicing factors such as Acinus and SRSF1, but none of the EJC-dependent splicing events depended on these factors. Second, Crabb and colleagues have shown that EJC deposition did not affect subsequent splicing kinetics in a reporter with two introns [58]. We also found that minigene vectors from EJC-regulated splicing events showed no difference in splicing with or without a pre-deposition of EJC (data not shown). Third, if the EJC acts as a classical splicing regulator such as Fox2 or Nova, then it should regulate alternative splicing in a positional-dependent manner [59], which can be visualised through a RNA-map [60]. However, RNA-map of eIF4A3 did not show significant changes in binding positions for the EJC-dependent inclusion and skipping events. Moreover, analysis of enhancers and silencer did not find any significant differences between the EJC-dependent inclusion and skipping exons, indicating that the EJC does not globally regulate alternative splicing as a classic splicing factor. Fourth, if the EJC deposition is required for the splicing of neighbouring introns as in *Drosophila*, EJC KD should cause more intron retention. However, there is a similar number of increase or decrease in intron retention in EJC KD cells, as described in *Drosophila* [29]. In addition, the EJC-dependent intron splicing events did not show specific characteristics compared with the EJC-dependent intron retention events.

The EJC plays a central role in the post-splicing life of mRNAs. Recently, EJC and SR proteins have been shown to participate in the formation of higher order mRNP particles and to contribute to the compaction of processed mRNAs [8]. It has become more and more clear that the packaging of pre-mRNAs and mRNAs can influence different steps of mRNA processing as well as transcription [1,3]. In yeast, connections between transcription and mRNA packaging has been clearly established, and its importance for quality control of mRNP has been revealed [61]. In mammals, coupling between RNA pol II transcription and pre-mRNA processing has also been established, and transcription has a clear impact on alternative splicing [3,62]. In this context, we tested whether some splicing events affected by EJC core downregulation could be coupled to transcription. We employed three different inhibitors of RNA pol II elongation (DRB, FP and CPT) and we observed that inhibition of RNA pol II elongation could rescue most of the EJC-dependent splicing changes. Indeed, even though the effect of CPT is variable, both DRB and FP consistently rescued the EJC-dependent splicing changes. Furthermore, by measuring the *in situ* transcription rate of specific genes, we found that Pol II transcription accelerates when the EJC proteins amount was reduced.

Two models have been proposed to explain how transcription can influence splicing: the ‘recruitment model’ and the ‘kinetic model’ [48,62]. In the kinetic model, variation of elongation rate controls the accessibility of competing splice sites in nascent RNA. A slower elongation would increase the time for splice site recognition or for binding of splicing regulators. Depending on the factors involved, variation in RNA pol II progression can favour the inclusion or the skipping of alternative exons while a faster elongation could reversely modulate these choices. We found that more exons were being kipped when EJC proteins were downregulated compared with exon inclusion, and that some previously considered as constitutive exons became excluded. By slowing down the transcription elongation rate, there is more time to recognise the EJC-dependent cassette exons, and to rescue exon skipping caused by EJC depletion. We also found that the EJC-dependent alternative exons generally have longer flanking introns, and this would allow more time for the alternative exons to be recognised by splicing enhancer or silencer to make the splicing decision before the downstream exon is transcribed. However, the EJC-dependent splicing events are not secondary effects caused by general changes in transcription rate, since not every exon regulated by transcription was affected by EJC KD. One example is the *CHD2* gene. The splicing changed significantly in response to transcription elongation inhibitors, but EJC KD did not show any effect. We also compared the EJC-dependent splicing events with the list of splicing changes in DRB and CPT treatment [47], and less than 15% of transcription-dependent splicing changes overlapped with the EJC-dependent events. We also found that the EJC-dependent intron retention events have significantly shorter exons with weaker splice sites. It has been shown recently that, in general, intron retention events were associated with increased GC content, reduced length and weaker 5′ and 3′ splice sites, and this was linked with localised stalling of Pol II and reduced availability of spliceosomes components [63]. For the EJC-dependent intron splicing events, the increased transcription elongation rate in EJC KD would mean less time between the 5′ and 3′ splice site recognition, and would favour intron retention. For the EJC-dependent intron retention events, the increased elongation rate would result in less Pol II pausing over retained introns and increase the splicing of these introns.

Taken together, our data reveal that the EJC participates in multiple alternative splicing events. At this stage, we believe that direct and indirect mechanisms could be responsible for this function of the EJC. The analysis of specific splicing events will certainly allow molecular links between the EJC and splicing regulation to be established. Interestingly, this study brings to light a potential connection between pre-mRNA synthesis and the EJC, suggesting that mRNP packaging may also look back on co-transcriptional processing in mammals.

## Materials and methods

### Antibodies and plasmids

Rabbit polyclonal anti-eIF4A3, anti-Y14 and anti-MLN51 are gifts from C. Tomasetto. Mouse monoclonal anti-eIF4A3 (3 F1) is a gift from G. Dreyfuss. Rabbit polyclonal anti-Upf1 and anti-Pol II (N20) are from Santa Cruz. Rabbit polyclonal anti-SRSF1, anti-SRSF2 and anti-SRSF7 are gifts from J. Stévenin. Rabbit polyclonal anti-Magoh is a gift from E. Izaurralde. Rabbit anti-FLAG is from Sigma. Rat monoclonal anti-S2P and anti-S5P are gifts from X. Darzacq. Mouse monoclonal anti-Pol II (Pol3/3) is a gift from O. Bensaude.

The original p3xFLAG-CMV-eIF4A3 was a gift from M. Moore. p3XFLAG-CMV-eIF4A3WT (siRNA-resistant) was created by site-directed mutagenesis (Invitrogen) for regions targeted by siRNA eIF4A3. The following primers were used: forward, 5′-GCTCAAAGAGGAAGACATGACGAAGGTAGAGTTCGAGACCAGCGAGGAGGTG-3′; reverse, 5′-CACCTCCTCGCTGGTCTCGAACTCTACCTTCGTCATGTCTTCCTCTTTGAGC-3′. The mutant eIF4A3 that does not form EJC (D401KE402R) was generated from the p3xFLAG-CMV-eIF4A3WT with the following primers: forward, 5′-CCACTCAGATTAAACGCATGCCGATGAACGTTGCTGATCTTATC-3′; reverse, 5′-AATAGTACTGCTCGATATCTCTGAGGATGCGGATG-3′.

### Cell culture and transfections

HeLa cells were maintained in DMEM (GIBCO) with 10% FCS (Life Technologies) and penicillin/streptomycin (Life Technologies). For siRNA knockdown, cells were transfected with 10 nM of siRNA using RNAiMax (Invitrogen) according to manufacturer’s protocol. Cells were harvested 48 h later. The following siRNA duplexes (Eurogentec) were used: siGFP, 5′-UGAAUUAGAUGGCGAUGUU-3′; sieIF4A3, 5′-AGACAUGACUAAAGUGGAA-3′; siY14, 5′-CGCUCUGUUGAAGGCUGGA-3′; siMLN51, 5′-GAUCGGAAGAAUCCAGCAU-3′; siUpf1, 5′-GAUGCAGUUCCGCUCCAUU-3′; siAcinus, 5′-GCUCGCUGCCCAAAUCAUU-3′; siSRSF1, 5′-CCAAGGACAUUGAGGACGU-3′; siSRSF2, 5′-AAUCCAGGUCGCGAUCGAA-3′; siSMG6, 5′-GCUGCAGGUUACUUACAAG-3′. For co-transfection of siRNA and plasmids, Lipofection 2000 (Invitrogen) was used. A total of 10 nM of siRNA was mixed together with 1 ug of p3xFLAG-CMV-eIF4A3WT or with 3 ug of p3xFLAG-CMV-eIF4A3Mut to obtain similar level of expression. Cells were collected 48 h after. For inhibition of Pol II elongation, cells were transfected with 10 nM siRNA for 30 h before adding DMSO (Sigma), 75 μM DRB (Sigma), 250 nM FP (Sigma) or 15 μM CPT (Sigma) for further 16 h.

### mRNA-seq and data analysis

For mRNA-seq, polyA+ mRNAs were extracted from HeLa cells treated with siRNA against GFP, eIF4A3, Y14, MLN51 and Upf1 using Illumina TruSeq RNA sample preparation kit (Illumina) according to manufacturer’s protocol. The fragmented mRNAs were sequenced using Illumina Hi-Seq 2000 single end sequencing with 51 nt length.

### Mapping of reads to the human genome

Raw reads that do not pass the Illumina quality filter were first discarded. The remaining mRNA-seq reads were mapped to the human genome (hg19) using TopHat v.2.08 [64]. Ensembl65 annotations were provided to TopHat (−G option). Alignments with reads that matches multiple positions on the genome were removed.

### Analysis of differential gene expression

From the mapped reads and the GTF annotation files (Ensembl65), we counted the number of reads for each gene using htseq-count [65]. To normalise the read counts and perform differential gene expression analysis, we used the DESeq package [33]. There were 24,978 genes with read counts. We chose a read coverage of 10 as a threshold in any of the KD conditions to ensure transcripts are reasonably expressed. The coverage was computed as the read length (51 bases) multiplied by the number of reads divided by the transcript length. This filter selected 10,881 genes. Then we filtered transcripts with significant expression changes using the cutoff of adjusted *P* value 0.05.

### Identification of differential exon usage

We use two different software to perform differential exon usage analysis: MISO [34] and DiffSplice [35].

### MISO

MISO estimates isoform expression (Ψ values, for ‘Percent Spliced Isoform’) and differential isoform expression for RNA-Seq data. We used the exon-centric analysis, which perform expression estimates at the alternative splicing event level. It provides confidence intervals for expression estimates and quantitative measures of differential expression (‘Bayes factors’). Since MISO does not handle replicate experiments, the program was run independently for each replicate (that is, compare GFPa vs 4A3a and GFPb vs 4A3b). The parameters used to filter differentially expressed events were: ∆Ψ >0.1, bayes-factor >5, number of skipping reads >10, number of inclusion reads >10, sum of inclusion and skipping reads >20.

### DiffSplice

DiffSplice identifies alternative splicing modules (ASMs) that correspond to genomic regions where alternative transcripts diverge. The ASMs are detected from a splice graph, which is built from RNA-seq reads. The method handles replicate experiments and does not depend on gene annotations, allowing the discovery of novel alternative splicing events. The parameters used for the splice graph construction were 5 for thresh_average_read_coverage_exon and 10 for thresh_average_read_coverage_intron. The thresholds for splice junction filtering (thresh_junction_filter_max_read_support, thresh_junction_filter_mean_read_support) were increased to 5 and thresh_junction_filter_num_samples_presence was set to 1. For the differential tests, the minimum value square root of JSD for significant differential transcription (thresh_sqrtJSD) was set to 0.1 and the false discovery rate was set to 1.

### Analysis of cassette exons with splicing changes

We focused on cassette exon events. We took the union of events with significant splicing change identified with MISO or DiffSplice with the threshold of ∆Ψ >0.1. Because DiffSplice only returned the end position of the upstream exon and the start position of the downstream exon, we retrieved the genomic coordinates of the upstream, cassette and downstream exon of the splicing event based on Ensembl65 annotations. We classified splicing events into two categories based on the direction of the change: events showing an increase in exon inclusion in EJC KD (ΔΨ < −0.1, −, 160 exons) and events showing an increase in exon skipping in EJC KD (ΔΨ >0.1, +, 291 exons). Furthermore, we classified events of the + category identified by DiffSplice into two categories: events corresponding to annotated alternative exons (ALT, 233 exons) and events corresponding to constitutive exons (exons having no annotation in the genome as alternative exons, CON, 58 exons).

As a control, we used two sets of exons: alternative exons from the MISO database that were not affected by eIF4A3 KD (Ctr, 991 exons) and constitutive exons from the genome (Ctr CON, 811 exons). Those exons were selected among expressed genes (read coverage more than 10). For the Ctr category, we filtered events with a splicing change less than 0.03 and with estimates of Ψ values included in (0.1;0.9) as predicted by MISO. For the Ctr CON category, we selected three successive constitutive exons based on Ensembl65 annotations, excluding the first and last exon of transcripts and allowing at most one triplet per gene.

Once exons with splicing changes have been identified, we look at possible correlations with different features. Computational analyses were performed using custom scripts in Python and R.

### Analysis of intron retention with splicing changes

We took the union of events with significant splicing change identified with MISO or DiffSplice with the threshold of |∆Ψ| >0.1. We retrieved the genomic coordinates of the exons flanking the intron retention events and the exons further upstream or downstream to define the flanking intron. We classified splicing events into two categories based on the direction of the change: events showing an increase in exon retention in EJC KD (ΔΨ <0.1, −, 134 exons) and events showing a decrease in exon retention in EJC KD (ΔΨ >0.1, +, 135 exons). As a control, we used intron retention events from the MISO database that were not affected by EJC KD with criteria as above (Ctr, 393 exons). Again, computational analyses were performed using custom scripts in Python and R.

### 5′ and 3′ splice sites strength

To compute a score for the 5′ and 3′ splice sites, we used the maximum entropy models for splice sites (MaxEntScan, [66]). The 5′ss scoring uses a 9-mer sequence (the last 3 nt of the exon and the first 6 nt of the downstream intron), while the 3′ss scoring uses a 23-mer sequence (the last 20 nt of the upstream intron and the first 3 nt of the exon).

### Analysis of splicing regulatory elements

For exonic splicing enhancers (ESE), we use the predictions of RESCUE-ESE [67], which identified 238 candidate hexamers in human genes. For exonic splicing silencers (ESS), we use the predictions of the fluorescence-activated screen for exonic splicing silencers (FAS-ESS, [68]), which identified 176 hexamers that occurred repeatedly in the screen. For intronic splicing enhancers (ISE), we use the predictions of the fluorescence-activated screen for intronic splicing enhancers (FAS-ISE, [69]), which identified 199 hexamers that occurred repeatedly in the screen. For intronic splicing silencers (ISS), we use the predictions of the fluorescence-activated screen for intronic splicing silencers (FAS-ISS, [70]), which yielded 102 decamers.

The frequency of ESE or ESS in the upstream, cassette and downstream exon was computed as the number of ESE or ESS in the exon normalised by the length of the exon. The frequency of ISE or ISS in the upstream and downstream intron was computed as the number of ISE or ISS in the intron divided by the length of the intron. To calculate the enrichment of each ESE, z score was calculated by comparing the frequency of ESE to the frequency of ESE in the same sequence randomised 100 times.

### Construction of RNA splicing map with eIF4A3 CLIP data

We plotted RNA splicing maps using the CLIP-seq data of eIF4A3 [7]. We analysed the CLIP-seq coverage in the cassette exon and upstream and downstream exon for the same sets of exons as described previously. We analysed 100 nt from the exon-intron border. The eIF4A3 CLIP-seq coverage was normalised by mRNA expression at the position (using the mRNA-seq data for siGFP), and the number of exons that cover this position. The normalised coverage was then represented as the fraction of reads at a position over the highest CLIP height of the analysed region (upstream, cassette and downstream exon). The height of CLIP reads at each position is the sum of all normalised coverage at that position for each category of exons.

### RNA extraction and RT-PCR analysis

Total RNA was extracted from cells using TRI reagent (Ambion) according to manufacturer’s protocol. The RNA was digested with 2U RNase-free DNase I (Ambion) for 30 min at 37°C before phenol extraction and precipitation. Reverse transcription was performed using 500 ng RNA with random primers and RevertAid reverse transcriptase (Fermentas) according to manufacturer’s protocol. For radioactive PCR analysis of alternative splicing, primer sets were designed across the constitutive exons. The list of primer sequences is shown in Additional file 8. To radioactively label the primers, 2.5 μM of each primer were incubated with ^32^P-γ-ATP and PNK (Fermentas) at 37°C for 30 min before purifying through a G6 column (Biorad). PCR reaction was performed with DreamTaq polymerase (Fermentas) using 1 μl of hot primers and 1 μl of cDNA for 25 cycles at 60°C annealing temperature. The 2x RNA Loading Dye (Ambion) were added to the PCR products, and were denatured at 95°C before resolving on the 8% denaturing polyacrylamide gel. Results were quantified with the Typhoon Imager (GE healthcare) and Image J software.

### Quantitative real-time PCR

Real-time PCR was performed using SYBR Select Master Mix (Life Technologies) on LightCycler (Roch). Primers for qPCR can be found in Additional file 14. The relatively amount of each RNA was calculated by the threshold cycle for each PCR product (Ct) in control and knockdown conditions, and compared with the house-keeping gene *GAPDH* (Glyceraldehyde-3-phosphate dehydrogenase).

### Immunoprecipitation and western blot analysis

HeLa cells were transfected with empty vectors or FLAG-tagged eIF4A3 for 48 h before collection. The cells were lysed in PXL buffer (1xPBS, 0.1% SDS, 0.5% NP-40, 0.5% Na deoxycholate) supplemented with protease inhibitor (Calbiochem), and incubated with RNAse A (Fermentas) and Turbo DNase (Ambion) for 10 min at 37°C. After centrifugation, the cell lysates were incubated with anit-FLAG beads (Sigma) for 2 h. After washing three times with 10 mM Tris–HCl, pH7.5, 150 mM NaCl, 2.5 mM MgCl_2_, 1% NP-40, the proteins were eluted by adding 1× elution buffer and heating at 50°C. The eluate was subjected to western blot analysis, and 1/15th of input was used.

For western blot analysis, cells were lysed in PXL buffer as described above. Equal amount of total protein were used, and separated by 6%, 10% or 12% of SDS-PAGE depending on the protein probed. The proteins were transferred to nitrocellulose membrane (Amersham), and blocked in 5% milk. The membrane was incubated with primary antibody (1:1,000 dilution except for Pol3/3, 1:500 dilution) for 2 h at room temperature before adding peroxidase-conjugated secondary antibodies (Thermo Scientific) for 1 h. The antibodies were detected by Femto ELC (Thermo Scientific) on a LAS400 machine (GE healthcare).

### RNA decay assay

HeLa cells were transfected with siRNA against GFP or eIF4A3 for 48 h. Cells were treated with 5 μg/ml of Actinomycin D (Sigma) and collected at various time points after the treatment. The total RNA was extracted using TRI reagent (Invitrogen), and RT-qPCR was performed as described above. *GAPDH* was used as a control gene. The relatively amount of mRNA in each sample was calculating by normalizing again *GAPDH*, and shown as a fraction of the mRNA level before Actinomycin D treatment.

### RNAPII transcription rate assay

The rates of RNA Pol II transcription were measured as previously described [56]. Briefly, HeLa cells were transfected with 10 nM siRNA against GFP, eIF4A3 or Y14 for 48 h. The cells were treated with 100 μM DRB in culture media for 3 h before washed with PBS and incubated with fresh media. Cells were collected at different time points after DRB removal and lysed in TRI reagent (Invitrogen) to extract total RNA. One microgram of total RNA was amplified using RevertAid reverse transcriptase (Fermentas), and the cDNA was used for real-time qPCR. *GAPDH* was used as a control gene (primers cross exon junction borders to detect mRNA). Each primer set was designed to across the exon-intron junction to detect the newly amplified pre-mRNA (Additional file 14). The relative amount of pre-mRNA at each time point was calculated by normalising against the *GAPDH* gene and shown as a fraction of the amount of pre-mRNA when cells were not treated with DRB.

### Data availability

High throughput sequencing data generated in this study has been submitted to NCBI’s Gene Expression Omnibus with the accession number GSE63091.

## Additional files

Additional file 1:
**mRNA-seq experiments in HeLa cells with EJC and UPf1 KD.** **(A)** Western blot of the KD efficiency for samples used in mRNA-seq experiments. **(B)** qPCR evaluation of the KD efficiency for samples used in mRNA-seq experiments. Data are shown as mean ± SD of triplicate experiments. ****P* <0.001, student *t*-test. **(C)** Pie-chart showing the percentage of uniquely mapped mRNA-seq reads for the different conditions. **(D)** Log plots showing the correlation of the number of uniquely mapped reads per genes between the two replicated mRNA-seq experiments for each KD condition. Pearson’s correlation coefficient (r^2^) for each comparison is indicated. **(E)** Log plots showing the correlation of the gene expression fold changes among the different KD conditions. Pearson’s correlation coefficient (r^2^) for each comparison is indicated. Additional file 2:
**Mapping information of mRNA-seq experiments.**
Additional file 3:
**List of genes with significant expression changes in any of the KD conditions.**
Additional file 4:
**Gene expression changes in EJC and Upf1 KD HeLa cells.** **(A)** Venn diagrams showing the overlap of genes with significant change in gene expression (padj <0.05) in each direction between eIF4A3, Y14 and MLN51 KD. **(B)** Venn diagrams showing the number of genes significantly changed in each direction for union of EJC KD and Upf1 KD. **(C)** RT-qPCR validation of predicted gene expression changes in eIF4A3 KD. The first five genes were predicted to be upregulated, and the next five genes were predicted to be downregulated in eIF4A3 KD. The last five genes were predicted to have no change in eIF4A3 KD. Data are shown as mean ± SD of triplicate experiments. ***P* <0.01; ****P* <0.001, student *t*-test. Additional file 5:
**Overlap of predicted alternative cassette exons for MISO and DiffSplice.** **(A)** Venn diagrams showing the overlap of significant splicing events identified with MISO between the two replicates of the eIF4A3, Y14 and MLN51 KD conditions, using a threshold of 0.1 for ΔΨ. **(B,C)** Venn diagrams showing the overlap of significant splicing events identified with MISO (B) or DiffSplice (C) between the eIF4A3, Y14 and MLN51 KD conditions (left panel) and between the EJC (union of events identified with eIF4A3, Y14 and MLN51) and UPF1 KD conditions (right panel), using a threshold of 0.1 for ΔΨ. The rates of RT-PCR validation for each category are shown. Additional file 6:
**Summary of alternative splicing events identified by MISO.**
Additional file 7:
**Summary of alternative splicing events identified by DiffSplice.**
Additional file 8:
**RT-PCR primers for validation of alternative splicing.**
Additional file 9:
**Validation of predicted cassette exons in EJC KDs.** **(A,B)** RT-PCR validation of cassette exon events predicted by MISO or DiffSplice, in different EJC core protein, Acinus, SR proteins and Upf1 KD cells. Both alternative (A) and constitutive (B) exons were chosen. The quantification of triplicate experiments are shown below as mean ± SD. **P* <0.05; ***P* <0.01; ****P* <0.001, one-way ANOVA. Additional file 10:
**List of alternative cassette splicing events identified by MISO and DiffSplice.**
Additional file 11:
**Validation of predicted alternative splicing changes.** **(A-C)** RT-PCR validation of A5SS (A), A3SS **(B)** and IR (C) events predicted by MISO. **(D)** RT-PCR validation of cassette exons that are changed in Upf1 KD. The quantification of triplicate experiments are shown below as mean ± SD. **P* <0.05; ***P* <0.01; ****P* <0.001, one-way ANOVA. Additional file 12:
**Alternative splicing patterns in eIF4A3 rescue experiments.** RT-PCR validation of candidate alternative splicing events in control and eIF4A3 KD cells overexpressed with siRNA-resistant eIF4A3 (WT) or a mutant that does not form EJC (Mut). The quantification of triplicate experiments are shown below as mean ± SD. **P* <0.05; ***P* <0.01; ****P* <0.001, one-way ANOVA. Additional file 13:
**EJC KD does not affect mRNA decay.** **(A)** RT-PCR of candidate alternative splicing events in control and NMD factor SMG6 KD cells, in parallel with eIF4A3 and UPF1 KD. The quantification of triplicate experiments are shown below as mean ± SD. ****P* <0.001, one-way ANOVA. **(B,C)** mRNA decay assay for different genes in control, eIF4A3 and Upf1 KD cells. A positive control (*MYC*) and a negative control (*DNM2*) are shown in (B), and the EJC-dependent alternative splicing genes are shown in (C). For alternative splicing genes, two sets of primers were used to amplify either all isoforms or inclusion-specific isoforms, and the schematic diagram is shown above. Data are shown as mean ± SEM of four independent experiments. Additional file 14:
**List of qPCR primers.**
Additional file 15:
**Expression changes of splicing factors.**
Additional file 16:
**Gene expression changes of other splicing factors.** **(A)** qPCR of gene expression level of SR proteins, ASAP proteins and other splicing factors in eIF4A3, Y14, MLN51 and Upf1 KD conditions. Data are shown as mean ± SD of triplicate experiments. **P* <0.05; ***P* <0.01; ****P* <0.001, one-way ANOVA. **(B)** Western blot showing the level of SRSF1, SRSF2 and SRSF7 proteins in EJC protein KD cells. GAPDH is used as a control. Additional file 17:
**Correlation between EJC-dependent transcript level and intron length.** **(A-C)** Lot plots showing the correlation between the gene expression fold change and the maximum intron length in the corresponding transcript for KD of eIF4A3 (A), Y14 **(B)** and MLN51 (C). All the genes have a read coverage of more than 10. Additional file 18:
**Characteristic of EJC-dependent cassette exons.** **(A-F)** Box plot comparing EJC-dependent exon inclusion (+), exon skipping (−) and control exons that have no splicing changes (Ctr). **(G-L)** Box plot comparing EJC-dependent constitutive exon inclusion (Con) with control constitutive exons (Ctr Con). The *P* value is calculated with Mann–Whitney-Wilcoxon Test, and is indicated. (A, G) Box plot of the sum of 5′ and 3′ splice site scores of the upstream and downstream introns. **(B,H)** Box plot of the exon length for the upstream exon, cassette exon and downstream exon. **(C,I)** Box plot of the frequency of ESE for upstream exon, cassette exon and downstream exon. **(D,J)** Box plot of the frequency of ESS for upstream exon, cassette exon and downstream exon. **(E,K)** Box plot of the frequency of ISS within the upstream and downstream introns. **(F,L)** Box plot of the exon length for upstream exon, cassette exon and downstream exon. Additional file 19:
**List of ESE hexamer enriched in upstream constitutive, alternative and downstream constitutive exons.**
Additional file 20:
**RNA map of EJC-dependent splicing events.** **(A)** RNA splicing map showing the binding of eIF4A3 in the upstream exon, cassette exon and downstream exon, 100 nt from exon-intron boarders. EJC-dependent alternative exon inclusion is shown as red, constitutive exon inclusion is shown as purple. The control alternative exons (above) and constitutive exons (below) that does not change in EJC KD are shown as grey. The CLIP reads were normalised to mRNA expression level and exon length, and the height of CLIP reads at a position is the sum of all normalised CLIP reads at that position for each category of exons. **(B)** RNA splicing map showing the binding of eIF4A3 in the flanking introns around the cassette exons, 150 nt from exon-intron boarders. Additional file 21:
**List of intron retention events changed identified by MISO and DiffSplice.**
Additional file 22:
**Characterisation of EJC-dependent intron retention events.** Box plot comparing EJC-dependent intron retention (+), intron splicing (−) with control exons that have no splicing changes (Ctr). **(A)** Boxplot of intron length for alternative intron and the flanking introns. **(B)** Boxplot of sum of 5′SS and 3′SS score for alternative and flanking introns. **(C)** Boxplot of ESE frequency for exons around the alternative and flanking introns. Additional file 23:
**RNA Pol II elongation is linked with EJC-dependent splicing.** **(A)** Western blot of control and eIF4A3 KD cells treated with Pol II elongation inhibitor. DRB, 75 μM; FP, 250 nM; CPT, 15 μM. GAPDH is used as a control. **(B)** RT-PCR of splicing pattern of a representative gene *HERC4* exon 25 treated with increasing amount of FP. **(C)** RT-PCR of alternative splicing pattern in control and eIF4A3 KD cells with different elongation inhibitors. The quantification of triplicate experiments are shown below as mean ± SD. **P* <0.05; ***P* <0.01; ****P* <0.001, one-way ANOVA. Additional file 24:
**RNA Pol II processivity assay in control and eIF4A3 and Y14 KD cells.** The amount of pre-mRNA after transcription inhibitor DRB removal at different times was shown for control gene *ITPR1* **(A)** and an EJC-dependent cassette exon *GLRX3* **(B)**. The gene structure is shown above each graph, and arrows indicating the exon-intron junctions analysed. Data are shown as mean ± SEM for four different experiments.
