# Supplementary figures and images for: Transcriptome-wide modulation of splicing by the exon junction complex
Source: Genome Biol. 2014 Dec 5;15(12):551. doi: 10.1186/s13059-014-0551-7 (PMC4268817; doi:10.1186/s13059-014-0551-7)

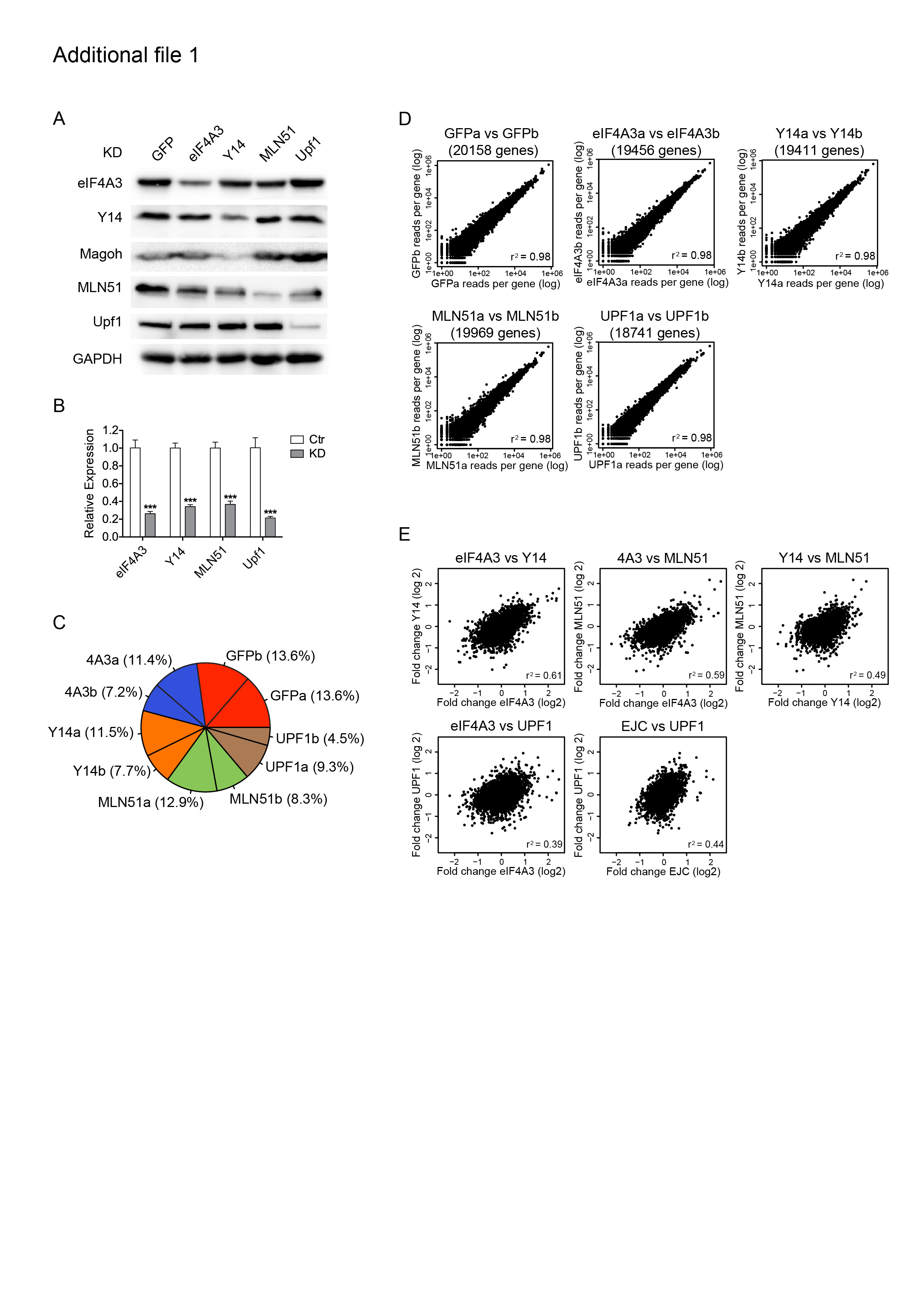

Supplement: Additional file 1: — mRNA-seq experiments in HeLa cells with EJC and UPf1 KD. (A) Western blot of the KD efficiency for samples used in mRNA-seq experiments. (B) qPCR evaluation of the KD efficiency for samples used in mRNA-seq experiments. Data are shown as mean ± SD of triplicate experiments. ***P <0.001, student t-test. (C) Pie-chart showing the percentage of uniquely mapped mRNA-seq reads for the different conditions. (D) Log plots showing the correlation of the number of uniquely mapped reads per genes between the two replicated mRNA-seq experiments for each KD condition. Pearson’s correlation coefficient (r2) for each comparison is indicated. (E) Log plots showing the correlation of the gene expression fold changes among the different KD conditions. Pearson’s correlation coefficient (r2) for each comparison is indicated. [file 13059_2014_551_MOESM1_ESM.jpg]

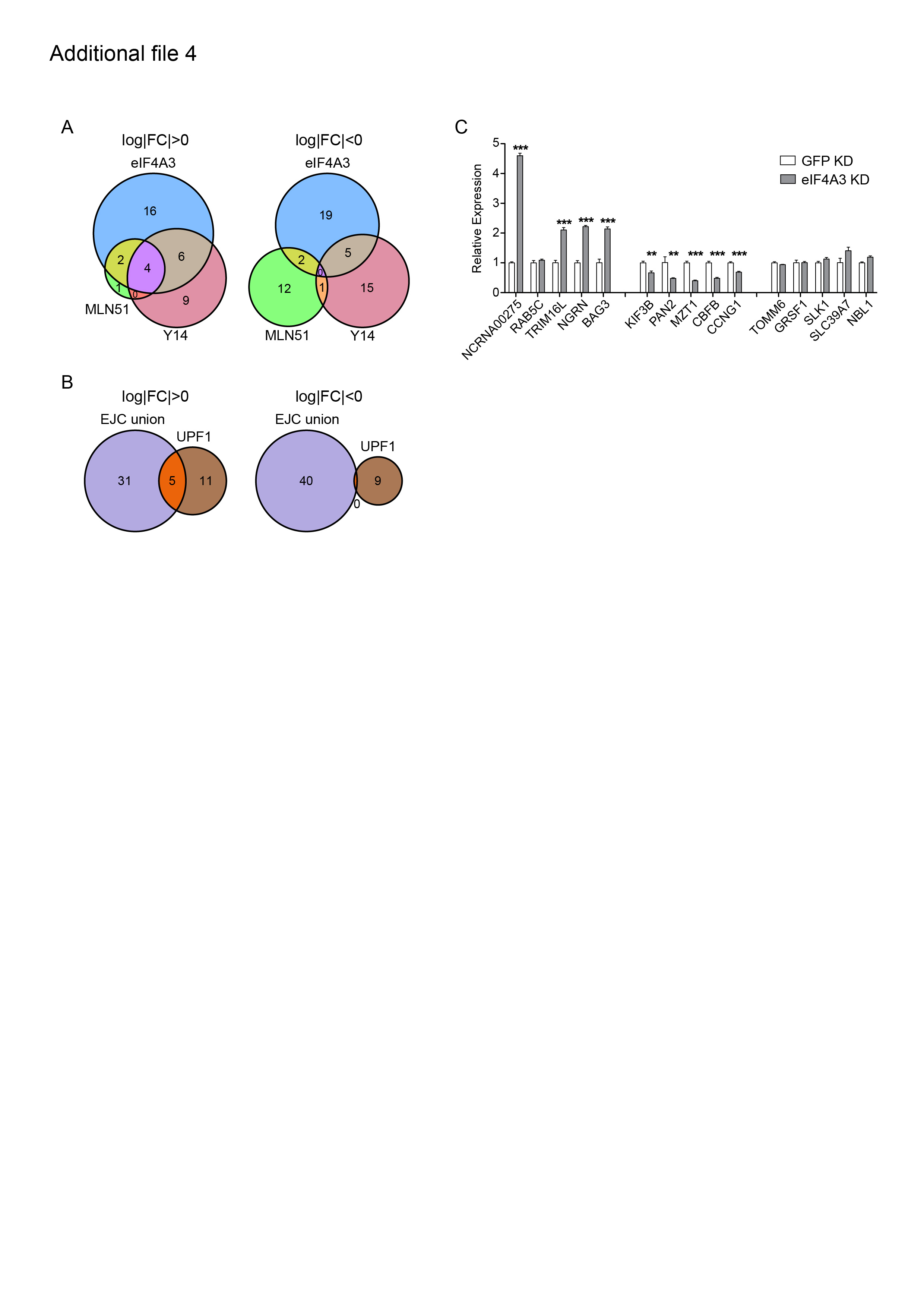

Supplement: Additional file 4: — Gene expression changes in EJC and Upf1 KD HeLa cells. (A) Venn diagrams showing the overlap of genes with significant change in gene expression (padj <0.05) in each direction between eIF4A3, Y14 and MLN51 KD. (B) Venn diagrams showing the number of genes significantly changed in each direction for union of EJC KD and Upf1 KD. (C) RT-qPCR validation of predicted gene expression changes in eIF4A3 KD. The first five genes were predicted to be upregulated, and the next five genes were predicted to be downregulated in eIF4A3 KD. The last five genes were predicted to have no change in eIF4A3 KD. Data are shown as mean ± SD of triplicate experiments. **P <0.01; ***P <0.001, student t-test. [file 13059_2014_551_MOESM4_ESM.jpg]

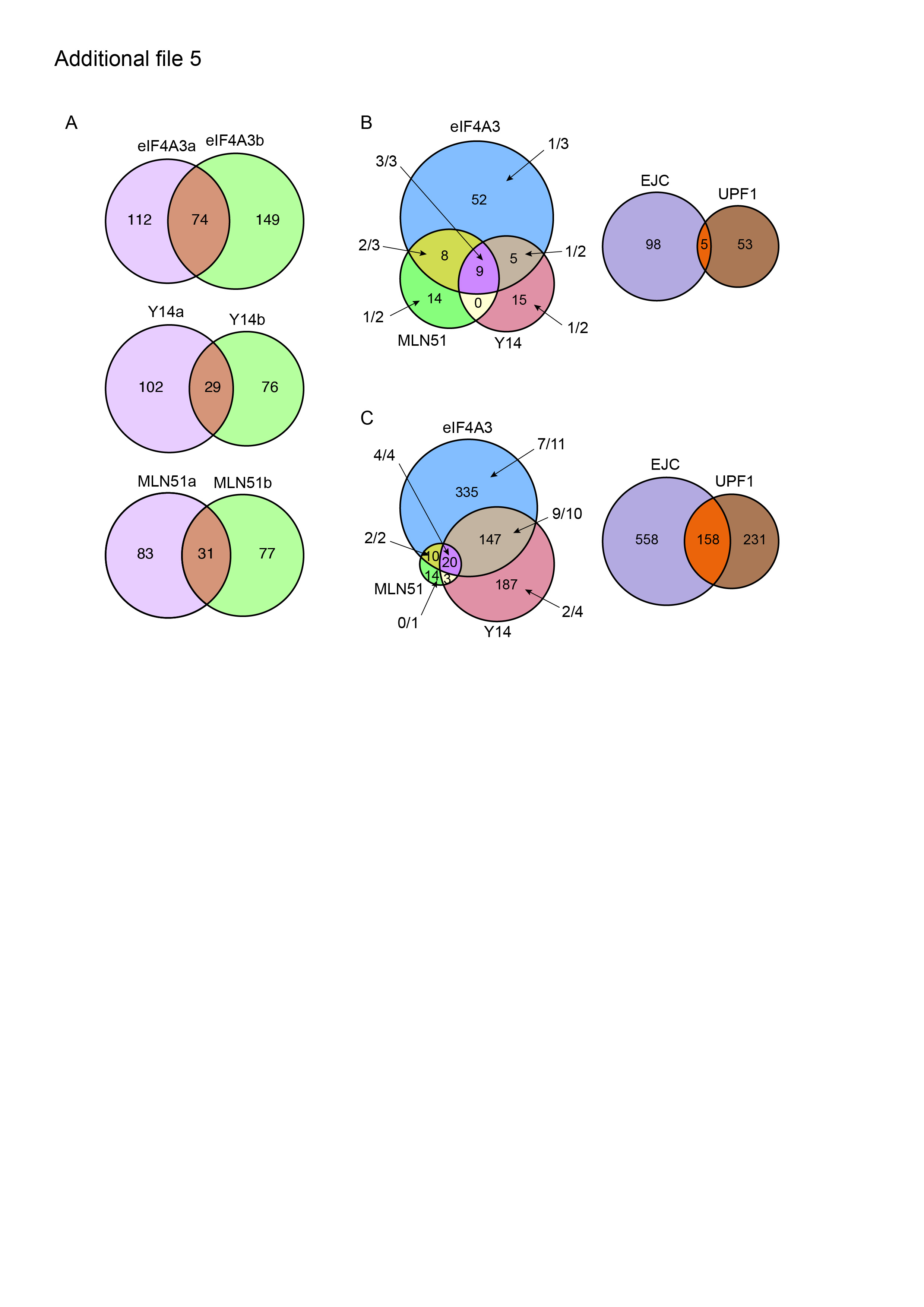

Supplement: Additional file 5: — Overlap of predicted alternative cassette exons for MISO and DiffSplice. (A) Venn diagrams showing the overlap of significant splicing events identified with MISO between the two replicates of the eIF4A3, Y14 and MLN51 KD conditions, using a threshold of 0.1 for ΔΨ. (B,C) Venn diagrams showing the overlap of significant splicing events identified with MISO (B) or DiffSplice (C) between the eIF4A3, Y14 and MLN51 KD conditions (left panel) and between the EJC (union of events identified with eIF4A3, Y14 and MLN51) and UPF1 KD conditions (right panel), using a threshold of 0.1 for ΔΨ. The rates of RT-PCR validation for each category are shown. [file 13059_2014_551_MOESM5_ESM.jpg]

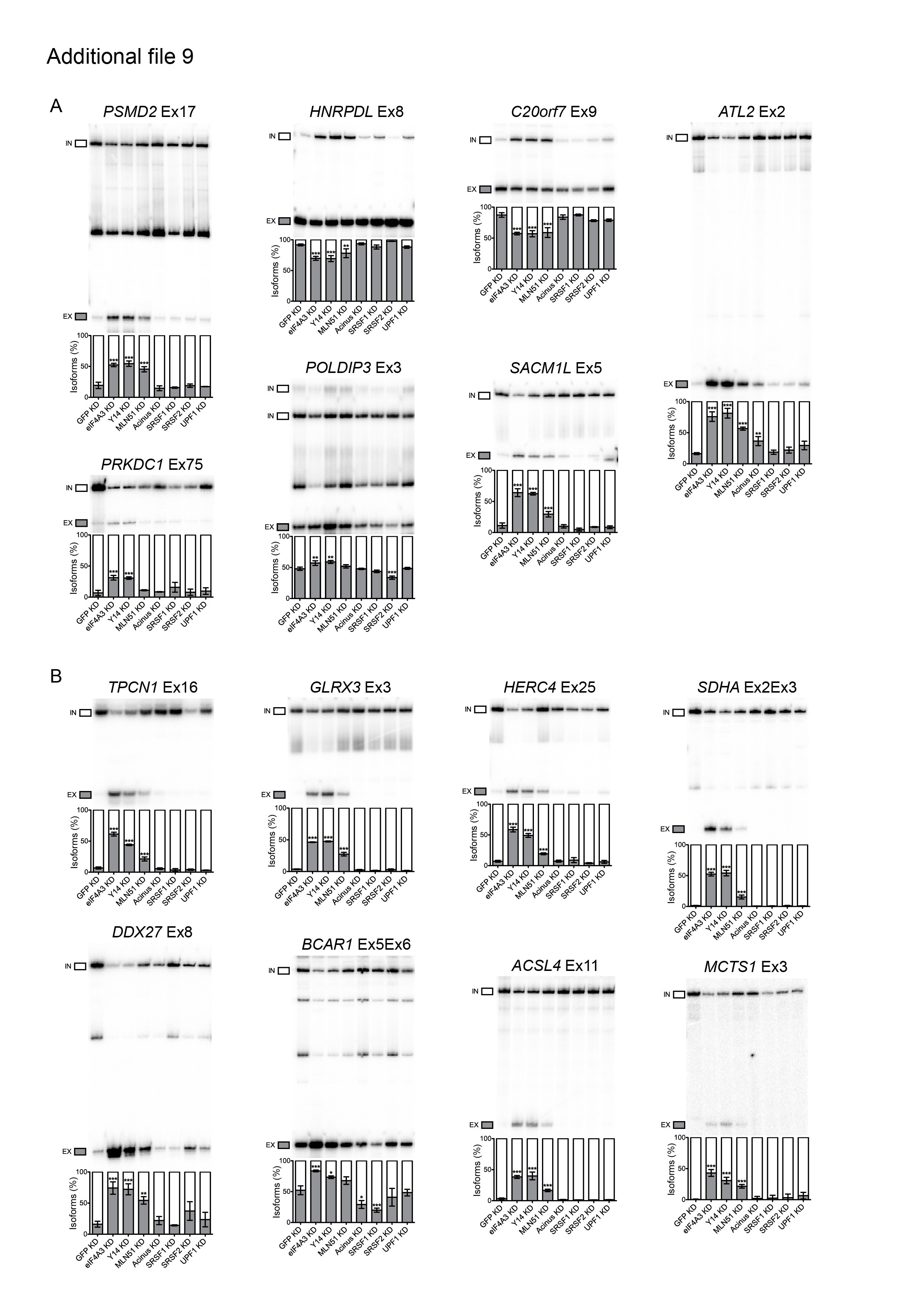

Supplement: Additional file 9: — Validation of predicted cassette exons in EJC KDs. (A,B) RT-PCR validation of cassette exon events predicted by MISO or DiffSplice, in different EJC core protein, Acinus, SR proteins and Upf1 KD cells. Both alternative (A) and constitutive (B) exons were chosen. The quantification of triplicate experiments are shown below as mean ± SD. *P <0.05; **P <0.01; ***P <0.001, one-way ANOVA. [file 13059_2014_551_MOESM9_ESM.jpg]

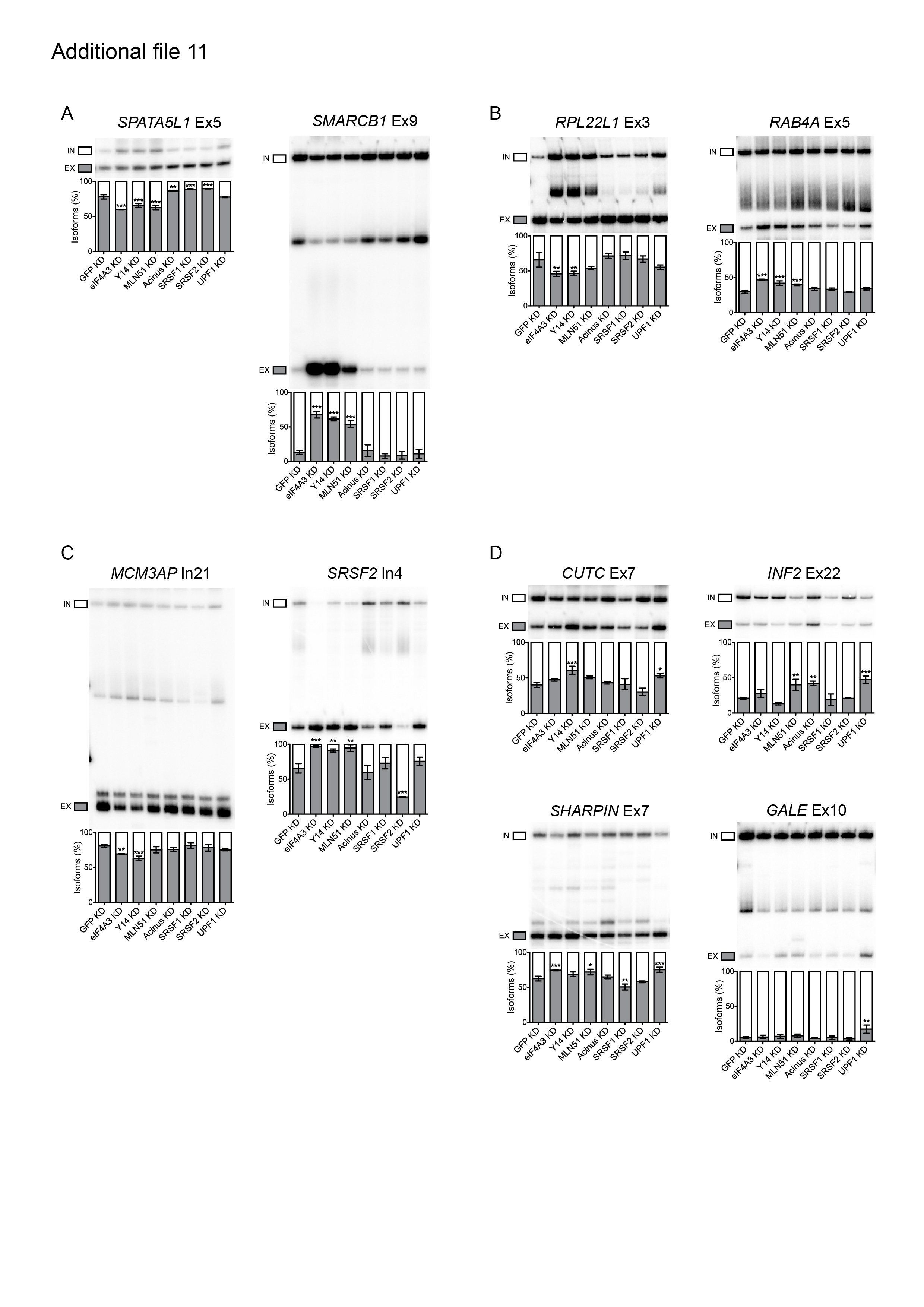

Supplement: Additional file 11: — Validation of predicted alternative splicing changes. (A-C) RT-PCR validation of A5SS (A), A3SS (B) and IR (C) events predicted by MISO. (D) RT-PCR validation of cassette exons that are changed in Upf1 KD. The quantification of triplicate experiments are shown below as mean ± SD. *P <0.05; **P <0.01; ***P <0.001, one-way ANOVA. [file 13059_2014_551_MOESM11_ESM.jpg]

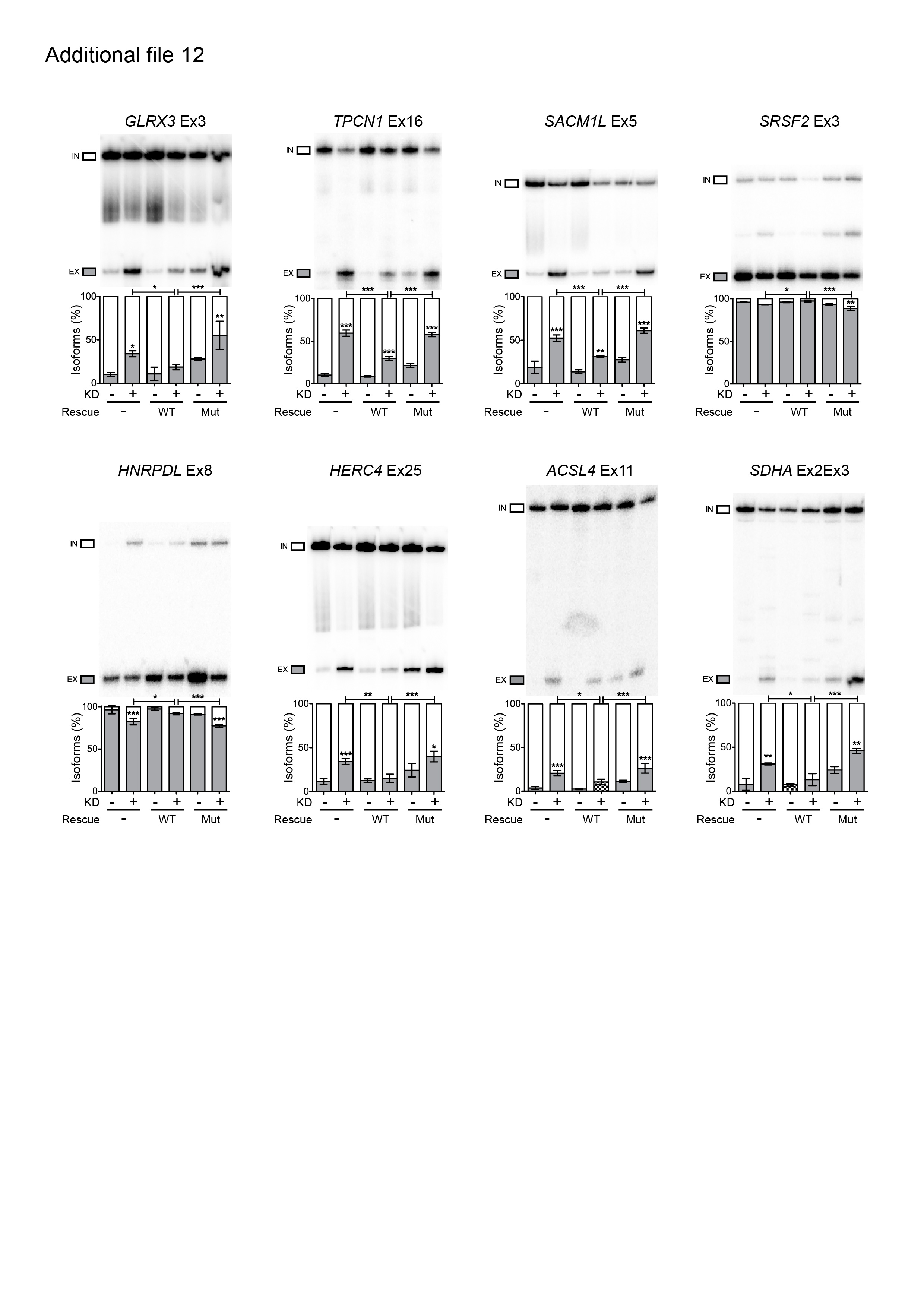

Supplement: Additional file 12: — Alternative splicing patterns in eIF4A3 rescue experiments. RT-PCR validation of candidate alternative splicing events in control and eIF4A3 KD cells overexpressed with siRNA-resistant eIF4A3 (WT) or a mutant that does not form EJC (Mut). The quantification of triplicate experiments are shown below as mean ± SD. *P <0.05; **P <0.01; ***P <0.001, one-way ANOVA. [file 13059_2014_551_MOESM12_ESM.jpg]

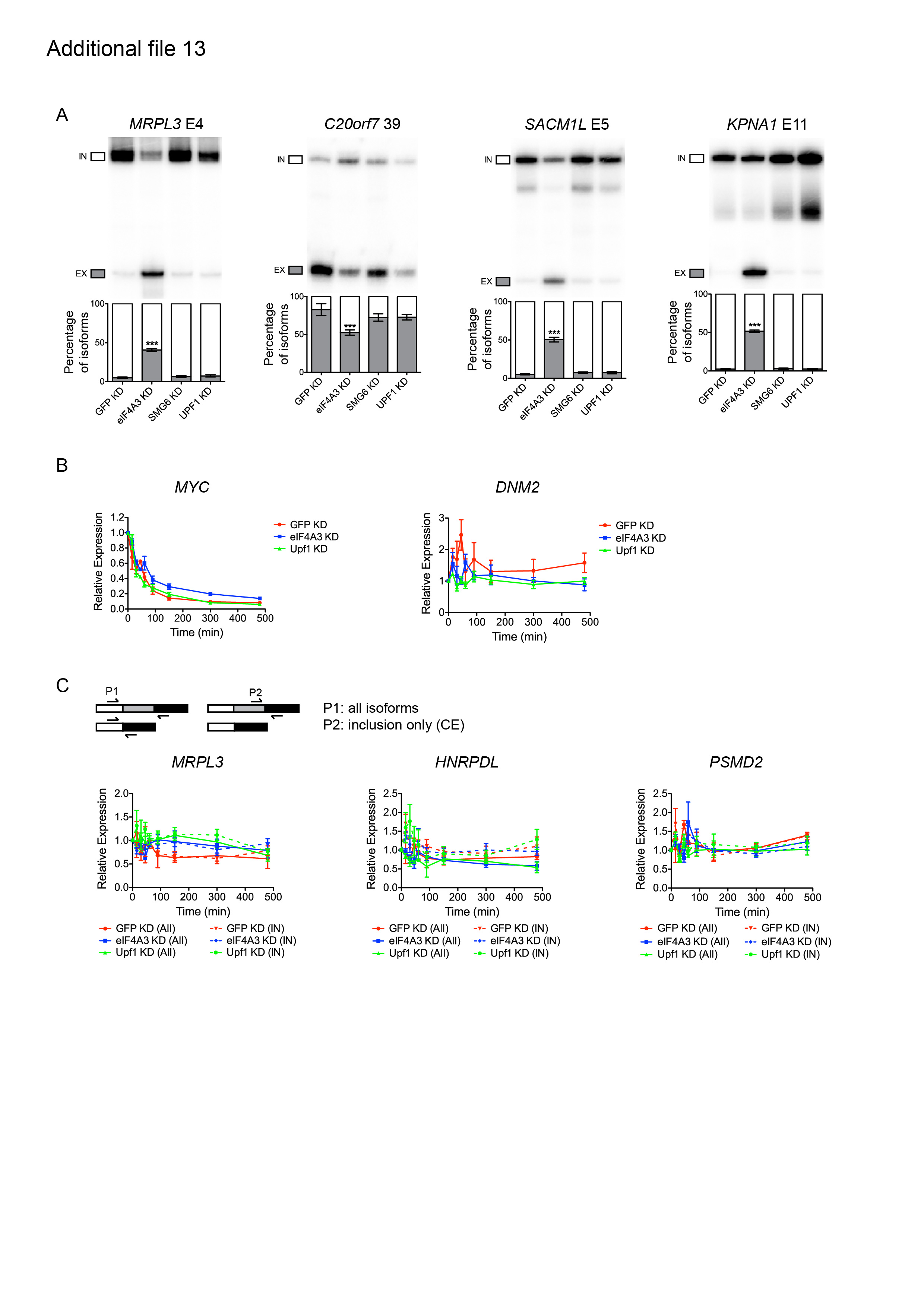

Supplement: Additional file 13: — EJC KD does not affect mRNA decay. (A) RT-PCR of candidate alternative splicing events in control and NMD factor SMG6 KD cells, in parallel with eIF4A3 and UPF1 KD. The quantification of triplicate experiments are shown below as mean ± SD. ***P <0.001, one-way ANOVA. (B,C) mRNA decay assay for different genes in control, eIF4A3 and Upf1 KD cells. A positive control (MYC) and a negative control (DNM2) are shown in (B), and the EJC-dependent alternative splicing genes are shown in (C). For alternative splicing genes, two sets of primers were used to amplify either all isoforms or inclusion-specific isoforms, and the schematic diagram is shown above. Data are shown as mean ± SEM of four independent experiments. [file 13059_2014_551_MOESM13_ESM.jpg]

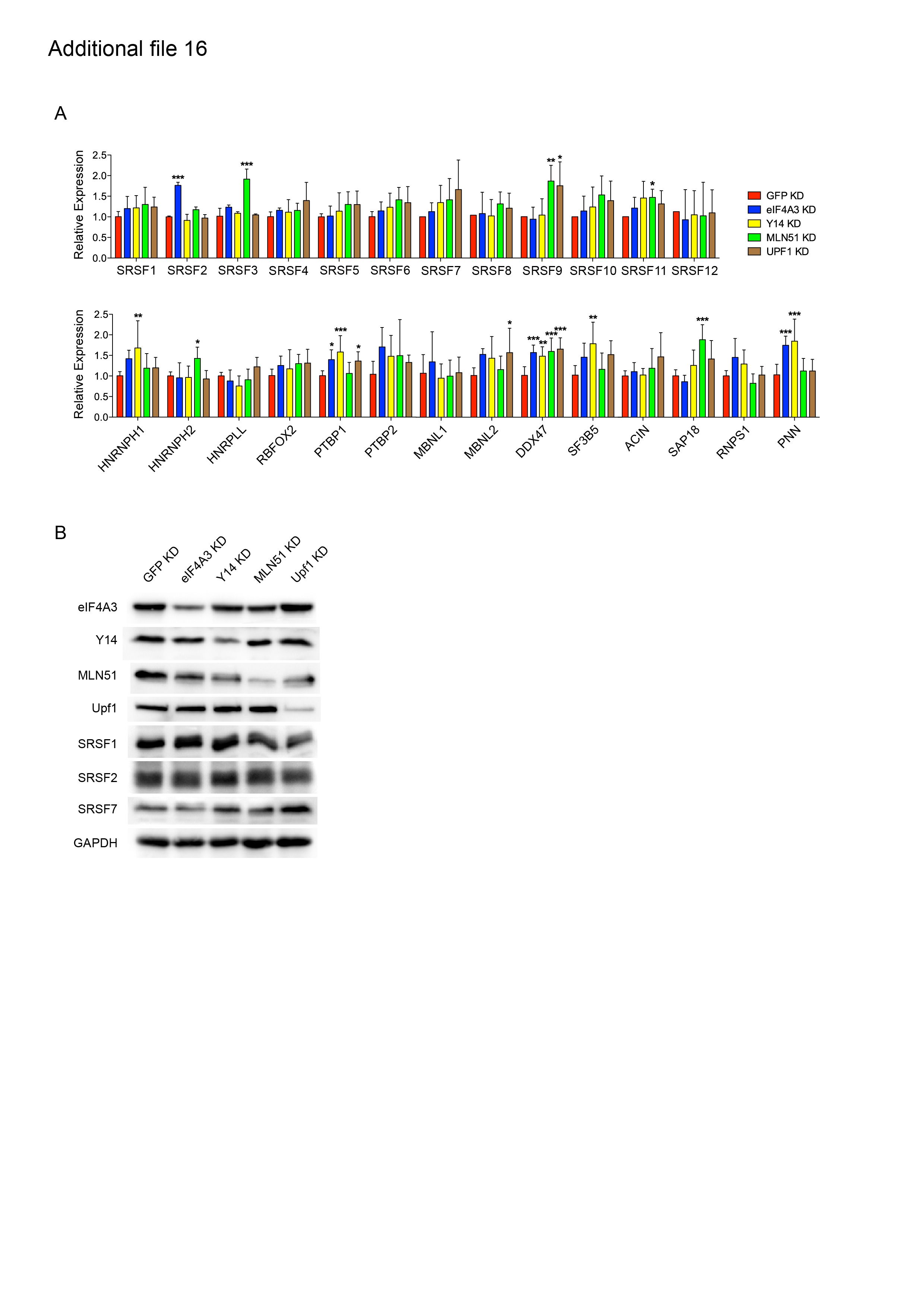

Supplement: Additional file 16: — Gene expression changes of other splicing factors. (A) qPCR of gene expression level of SR proteins, ASAP proteins and other splicing factors in eIF4A3, Y14, MLN51 and Upf1 KD conditions. Data are shown as mean ± SD of triplicate experiments. *P <0.05; **P <0.01; ***P <0.001, one-way ANOVA. (B) Western blot showing the level of SRSF1, SRSF2 and SRSF7 proteins in EJC protein KD cells. GAPDH is used as a control. [file 13059_2014_551_MOESM16_ESM.jpg]

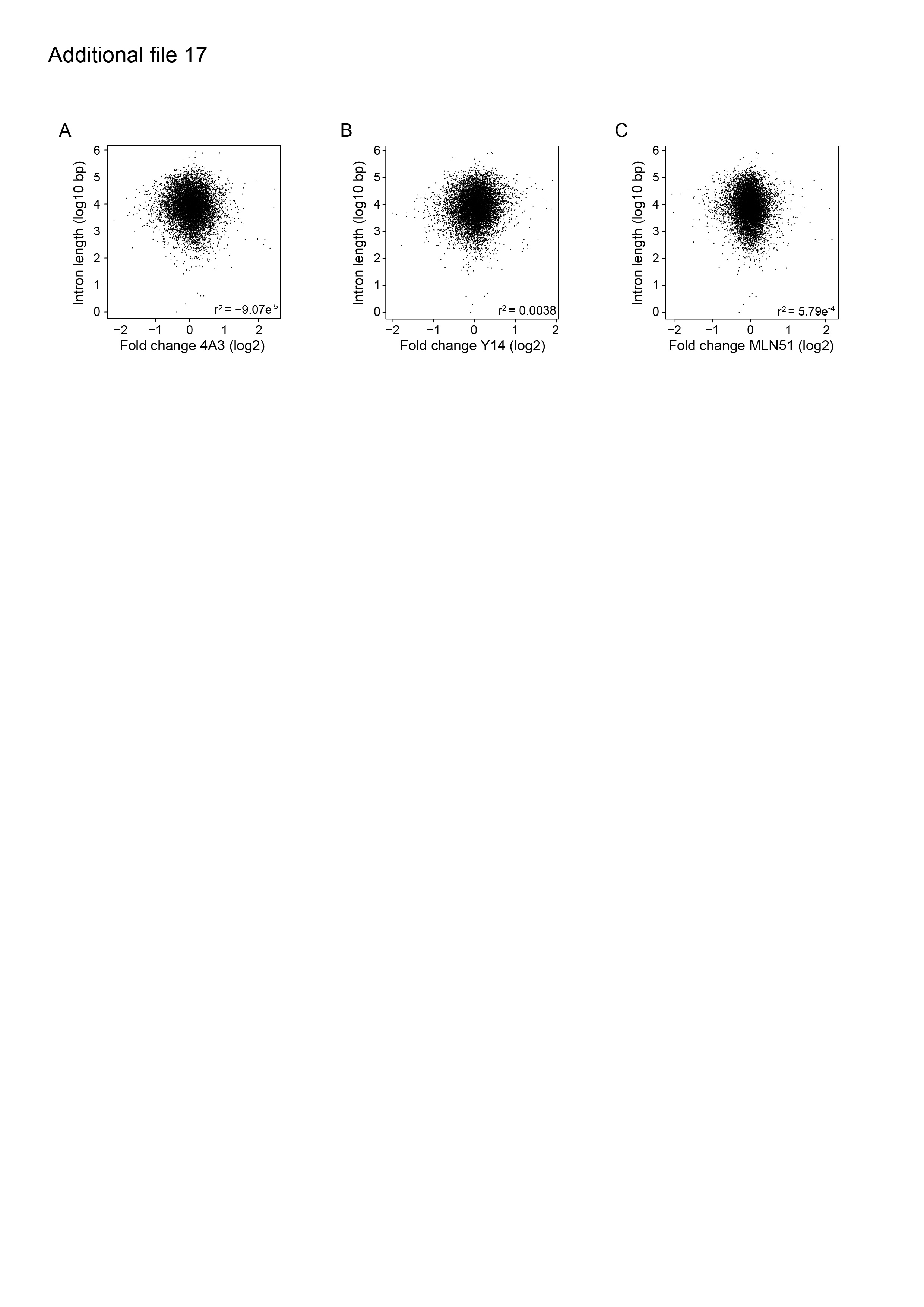

Supplement: Additional file 17: — Correlation between EJC-dependent transcript level and intron length. (A-C) Lot plots showing the correlation between the gene expression fold change and the maximum intron length in the corresponding transcript for KD of eIF4A3 (A), Y14 (B) and MLN51 (C). All the genes have a read coverage of more than 10. [file 13059_2014_551_MOESM17_ESM.jpg]

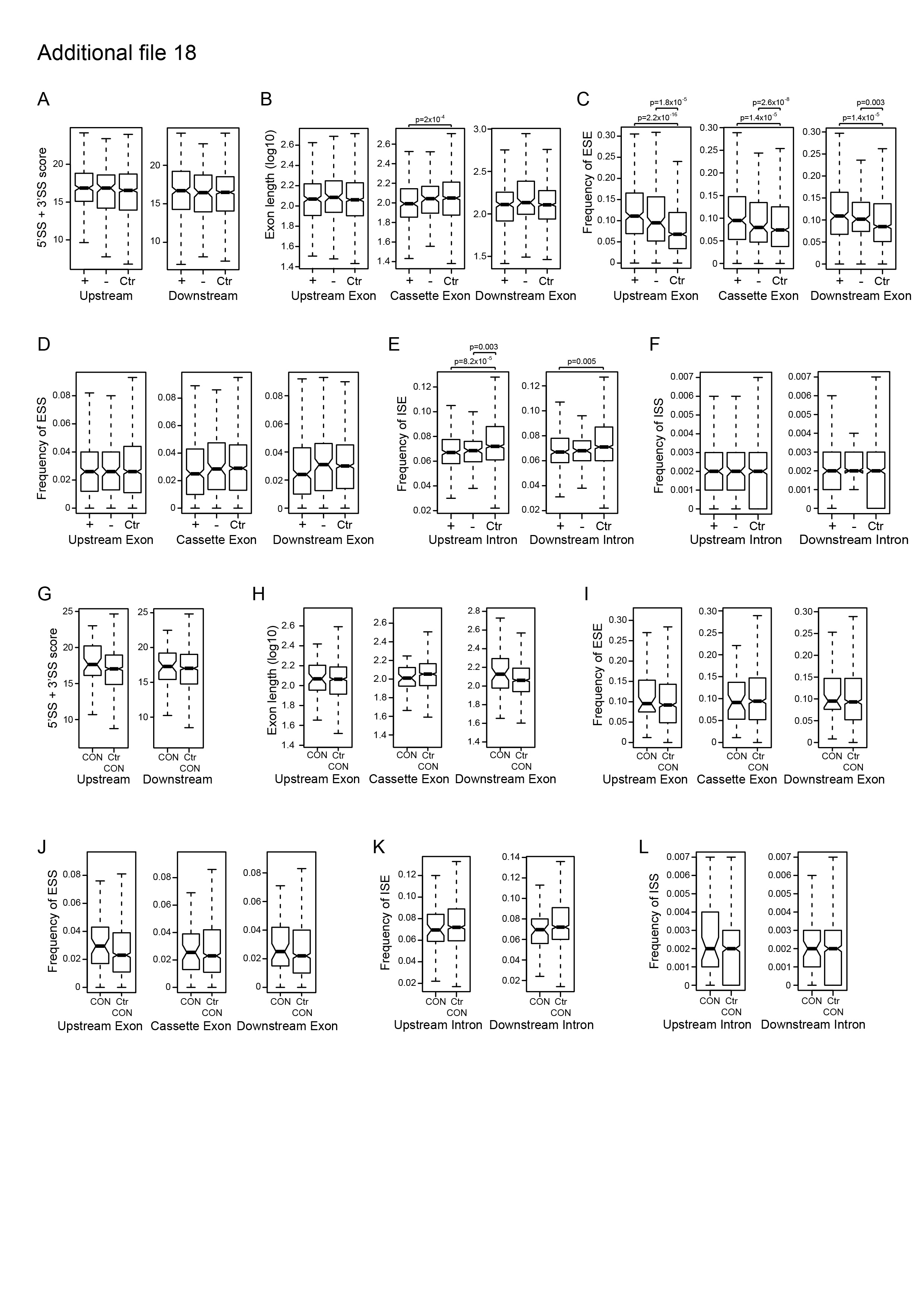

Supplement: Additional file 18: — Characteristic of EJC-dependent cassette exons. (A-F) Box plot comparing EJC-dependent exon inclusion (+), exon skipping (−) and control exons that have no splicing changes (Ctr). (G-L) Box plot comparing EJC-dependent constitutive exon inclusion (Con) with control constitutive exons (Ctr Con). The P value is calculated with Mann–Whitney-Wilcoxon Test, and is indicated. (A, G) Box plot of the sum of 5′ and 3′ splice site scores of the upstream and downstream introns. (B,H) Box plot of the exon length for the upstream exon, cassette exon and downstream exon. (C,I) Box plot of the frequency of ESE for upstream exon, cassette exon and downstream exon. (D,J) Box plot of the frequency of ESS for upstream exon, cassette exon and downstream exon. (E,K) Box plot of the frequency of ISS within the upstream and downstream introns. (F,L) Box plot of the exon length for upstream exon, cassette exon and downstream exon. [file 13059_2014_551_MOESM18_ESM.jpg]

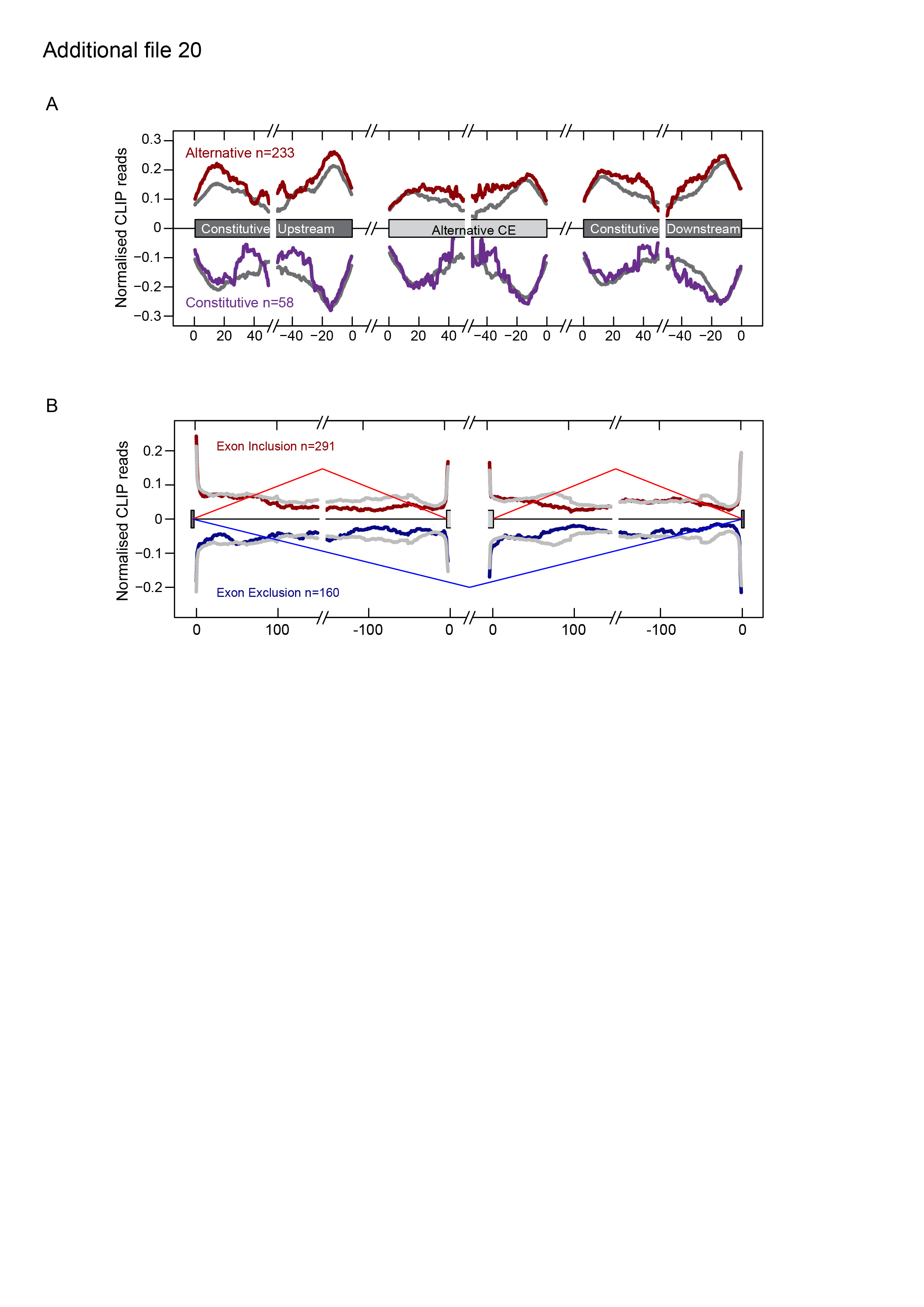

Supplement: Additional file 20: — RNA map of EJC-dependent splicing events. (A) RNA splicing map showing the binding of eIF4A3 in the upstream exon, cassette exon and downstream exon, 100 nt from exon-intron boarders. EJC-dependent alternative exon inclusion is shown as red, constitutive exon inclusion is shown as purple. The control alternative exons (above) and constitutive exons (below) that does not change in EJC KD are shown as grey. The CLIP reads were normalised to mRNA expression level and exon length, and the height of CLIP reads at a position is the sum of all normalised CLIP reads at that position for each category of exons. (B) RNA splicing map showing the binding of eIF4A3 in the flanking introns around the cassette exons, 150 nt from exon-intron boarders. [file 13059_2014_551_MOESM20_ESM.jpg]

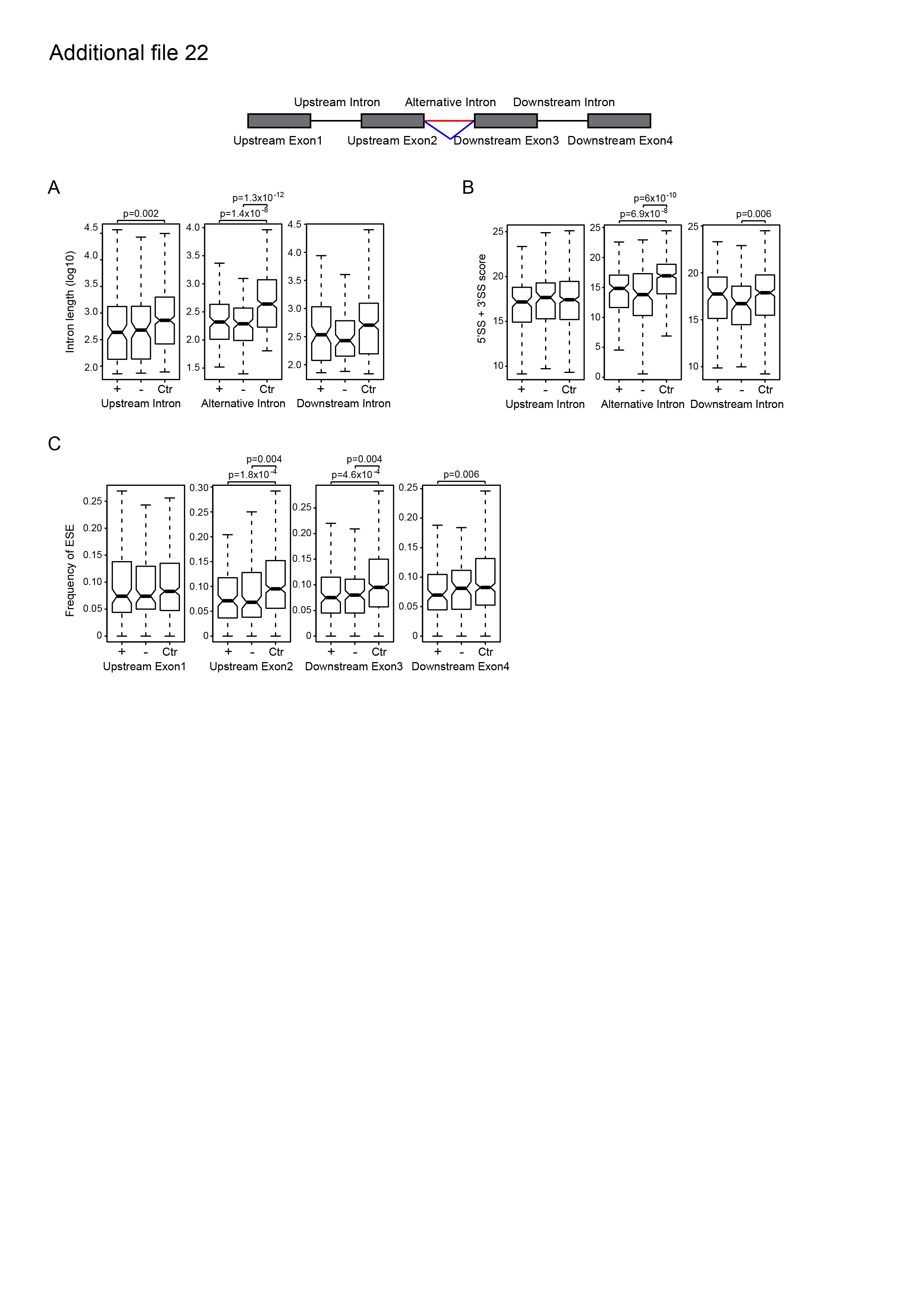

Supplement: Additional file 22: — Characterisation of EJC-dependent intron retention events. Box plot comparing EJC-dependent intron retention (+), intron splicing (−) with control exons that have no splicing changes (Ctr). (A) Boxplot of intron length for alternative intron and the flanking introns. (B) Boxplot of sum of 5′SS and 3′SS score for alternative and flanking introns. (C) Boxplot of ESE frequency for exons around the alternative and flanking introns. [file 13059_2014_551_MOESM22_ESM.jpg]

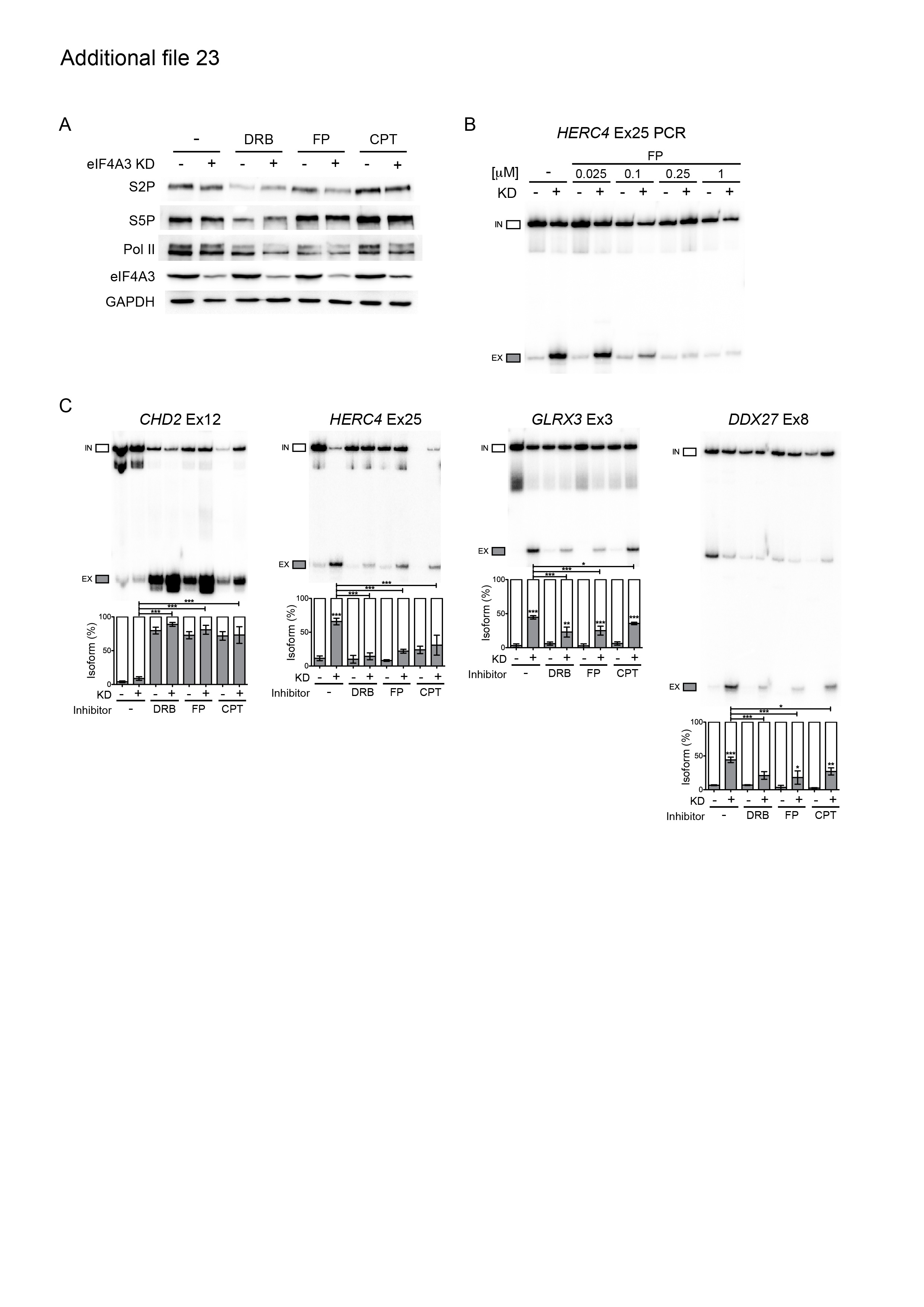

Supplement: Additional file 23: — RNA Pol II elongation is linked with EJC-dependent splicing. (A) Western blot of control and eIF4A3 KD cells treated with Pol II elongation inhibitor. DRB, 75 μM; FP, 250 nM; CPT, 15 μM. GAPDH is used as a control. (B) RT-PCR of splicing pattern of a representative gene HERC4 exon 25 treated with increasing amount of FP. (C) RT-PCR of alternative splicing pattern in control and eIF4A3 KD cells with different elongation inhibitors. The quantification of triplicate experiments are shown below as mean ± SD. *P <0.05; **P <0.01; ***P <0.001, one-way ANOVA. [file 13059_2014_551_MOESM23_ESM.jpg]

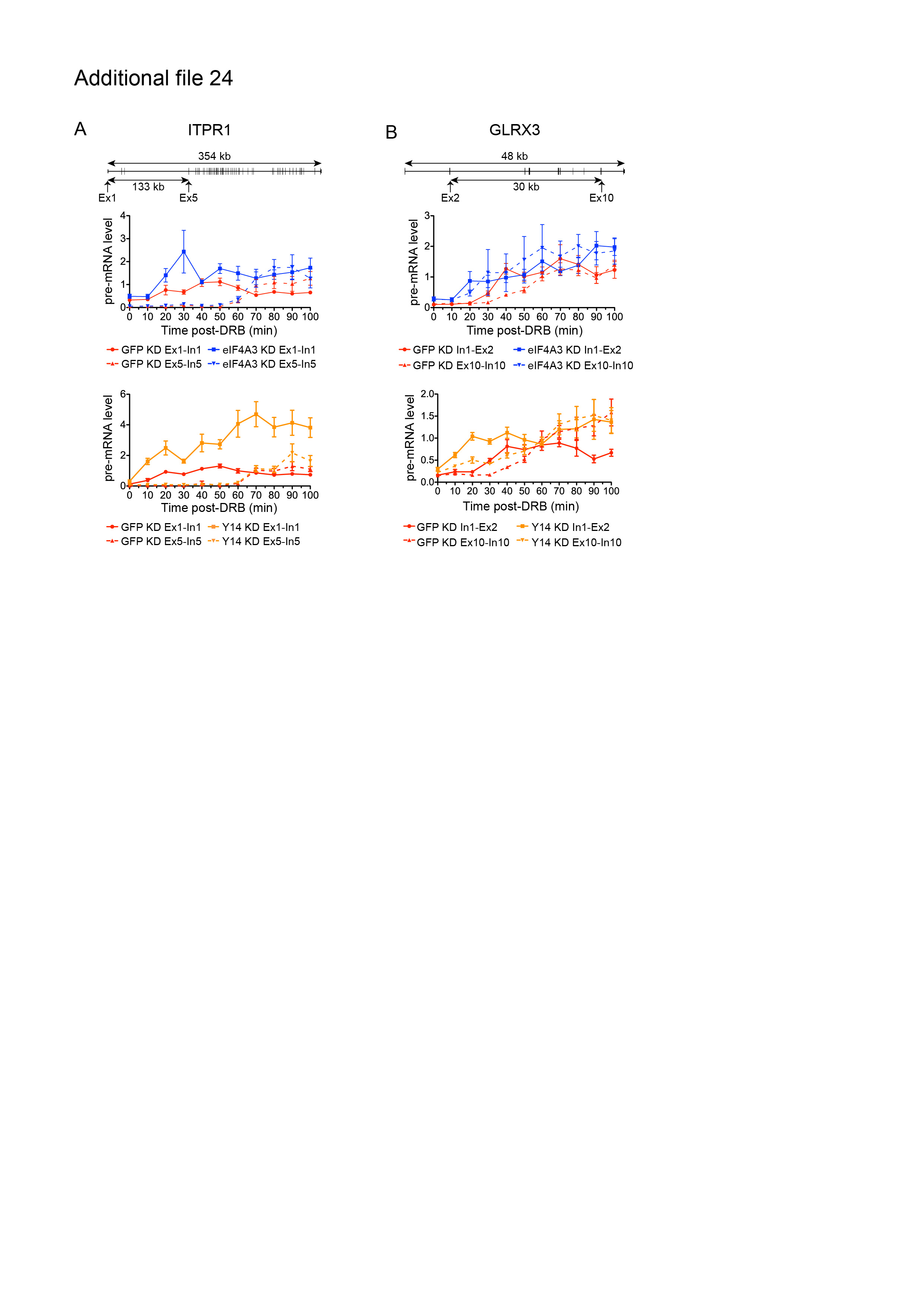

Supplement: Additional file 24: — RNA Pol II processivity assay in control and eIF4A3 and Y14 KD cells. The amount of pre-mRNA after transcription inhibitor DRB removal at different times was shown for control gene ITPR1 (A) and an EJC-dependent cassette exon GLRX3 (B). The gene structure is shown above each graph, and arrows indicating the exon-intron junctions analysed. Data are shown as mean ± SEM for four different experiments. [file 13059_2014_551_MOESM24_ESM.jpg]
