# Supplementary material for: Transcriptome-wide modulation of splicing by the exon junction complex
Source: Genome Biol. 2014 Dec 5;15(12):551. doi: 10.1186/s13059-014-0551-7 (PMC4268817; doi:10.1186/s13059-014-0551-7)
Supplement: Additional file 2: — Mapping information of mRNA-seq experiments. [file 13059_2014_551_MOESM2_ESM.pdf]

Additional file 2. Mapping information of mRNA-seq experiments.

Dataset	GFPa	GFPb	eIF4A3a	eIF4A3b	Y14a	Y14b	MLN51a	MLN51b	Upf1a	Upf1b
Raw reads	66 282 331	59 986 235	56 384 890	31 000 101	54 652 429	33 688 760	63 050 056	36 604 406	40 315 634	19 443 518
Quality filtered reads	54 670 947 (82%)	54 088 976 (92%)	46 003 944 (82%)	28 516 818 (92%)	45 862 214 (84%)	31 037 137 (92%)	51 758 633 (82%)	33 651 841 (92%)	37 050 820 (92%)	17 957 810 (92%)
Tophat unique reads	46 978 700 (86%)	46 860 274 (87%)	39 373 385 (86%)	24 822 271 (87%)	39 547 567 (86%)	26 680 610 (86%)	44 378 492 (86%)	28 747 133 (85%)	32 095 191 (87%)	15 595 789 (87%)
