# Supplementary material for: Transcriptome-wide modulation of splicing by the exon junction complex
Source: Genome Biol. 2014 Dec 5;15(12):551. doi: 10.1186/s13059-014-0551-7 (PMC4268817; doi:10.1186/s13059-014-0551-7)
Supplement: Additional file 6: — Summary of alternative splicing events identified by MISO. [file 13059_2014_551_MOESM6_ESM.pdf]

Additional file 6. Alternative splicing events identified by MISO.

Comparison	SE	RI	MXE	A5SS	A3SS	AFE	ALE	Tandem UTR
GFPa/eIF4A3a	186	83	11	68	62	208	257	0
GFPb/eIF4A3b	223	123	14	87	83	470	306	0
GFPa/Y14a	131	34	3	36	42	114	156	0
GFPb/Y14b	105	56	5	34	31	156	166	0
GFPa/MLN51a	114	47	9	33	32	312	213	0
GFPb/MLN51b	108	66	10	39	37	182	111	0
GFPb/Upf1a	168	79	7	27	37	80	66	0
GFPb/Upf1b	140	89	8	29	38	1021	897	0

SE - Cassette Exon; RI - Intron Retention; MXE - Mutually Exclusive; A5SS - Alternative 5' splice site; A3SS - Alternative 3' splice site
AFE - Alternative First Exon; ALE - Alternative Last Exon.
