# Supplementary material for: Transcriptome-wide modulation of splicing by the exon junction complex
Source: Genome Biol. 2014 Dec 5;15(12):551. doi: 10.1186/s13059-014-0551-7 (PMC4268817; doi:10.1186/s13059-014-0551-7)
Supplement: Additional file 7: — Summary of alternative splicing events identified by DiffSplice. [file 13059_2014_551_MOESM7_ESM.pdf]

Additional file 7. Alternative splicing events identified by Diffsplice.

Comparison	Significant ASM	Exon skipping	Intron retention	Alternative start/end	Uncataloged	Mutually exclusive
GFP/eIF4A3	1522 (27%)	512	198	174	638	0
GFP/Y14	1034 (19%)	357	124	95	457	1
GFP/MLN51	113 (2%)	47	13	4	49	0
GFP/Upf1	1115 (23%)	389	196	115	415	0
